# Light-Mediated Interconversion between a Foldamer and a Self-Replicator

**DOI:** 10.1021/jacs.4c09114

**Published:** 2024-11-26

**Authors:** Yulong Jin, Pradeep K. Mandal, Juntian Wu, Armin Kiani, Rui Zhao, Ivan Huc, Sijbren Otto

**Affiliations:** † Beijing National Laboratory for Molecular Sciences, CAS Key Laboratory of Analytical Chemistry for Living Biosystems, Institute of Chemistry, Chinese Academy of Sciences, 100190 Beijing, China; ‡ Department of Pharmacy, Ludwig-Maximilians-Universität München, 81377 Munich, Germany; § Centre for Systems Chemistry, Stratingh Institute, University of Groningen, 9747 AG Groningen, The Netherlands; ∥ State Key Laboratory of Chemical Resource Engineering, Beijing Advanced Innovation Center for Soft Matter Science and Engineering, College of Chemistry, Beijing University of Chemical Technology, 100029 Beijing, China

## Abstract

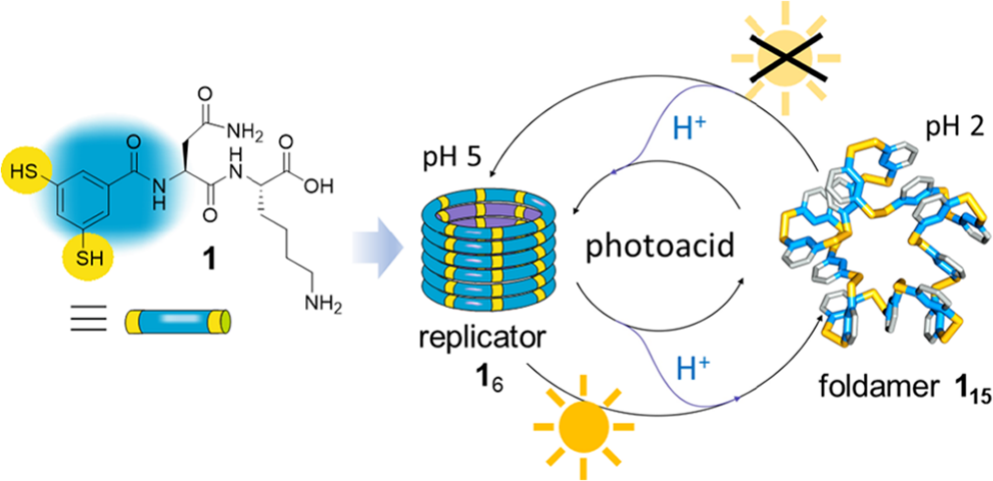

Self-replicating molecules and well-defined folded macromolecules are of great significance in the emergence and evolution of life. How they may interconnect and affect each other remains largely elusive. Here, we demonstrate an abiotic system where a single building block can oligomerize to yield either a self-replicating molecule or a foldamer. Specifically, agitation of a disulfide-based dynamic combinatorial library at moderately elevated pH channels it selectively into a self-replicating hexamer assembled into fibers, after passing through a period where a 15-subunit macrocyclic foldamer existed transiently. Without mechanoagitation or at lower pH, the formation of hexamer fiber is suppressed, resulting in the accumulation of the 15mer foldamer. Foldamer and self-replicator can be interconverted in response to external stimuli, including agitation and a change in pH. Furthermore, upon the addition of a photoacid, the pH of the medium can be controlled by irradiation, driving the switching between replicator and foldamer and allowing a dissipative out-of-equilibrium state to be accessed, using light as a source of energy.

## Introduction

Molecules that can self-replicate or fold are of great significance in the emergence and early evolution of life.^1−3^ In nature, replication is done with nucleic acids, while folded molecules like proteins exert other diverse functions, such as molecular recognition and catalysis. Through the central dogma of molecular biology, the information stored in nucleic acids is translated into proteins. However, in primitive life, the complex biochemical machinery that currently mediates transcription and translation was unlikely to be present, raising the question of how folded and self-replicating oligomers can become connected.

Before addressing this question, let us briefly survey the largely unconnected work on synthetic self-replicating molecules and synthetic foldamers. Traditionally, both replicators^1,4−10^ and foldamers^11−19^ have developed based on rational design, often inspired by their biological counterparts. More recently, it has been shown that both classes of molecules can also emerge spontaneously from dynamic combinatorial libraries (DCLs).^20^ In this approach, the noncovalent interactions that form intramolecularly within a specific library member, or intermolecularly between library members, drive the formation of specific foldamers^21,22^ or self-replicators,^23,24^ respectively, which often have structures that would not be readily accessible by design.

Whether foldamers or self-replicators emerge from dynamic combinatorial libraries is determined by whether noncovalent interactions form intra- or intermolecularly. This means that both compound classes can have a lot in common. In fact, we previously observed that both can form from the same building blocks.^25,26^ However, at equilibrium, only one of them (often the self-replicator) will remain. Hence, allowing access to both will require a means of maintaining the system away from equilibrium.

Inspired by nature, chemists are increasingly interested in developing approaches to maintain synthetic chemical systems away from equilibrium, driven by chemical fuels,^27−31^ electrically,^32^ or through photoirradiation.^33,34^

Here, we demonstrate the autonomous formation of a self-replicator and a foldamer from the same building block as well as the efficient interconversion between them by applying mechanical energy or by a pH change. We also show that upon using a photoacid, photoirradiation can maintain the system away from equilibrium, driving the conversion of the self-replicator to the foldamer. The foldamer only persists as long as irradiation is continued; as soon as it is switched off, the replicator regains dominance.

## Results and Discussion

### Building Block Design and Mechanoresponsiveness of the Dynamic Combinatorial Library

**Figure 1:**
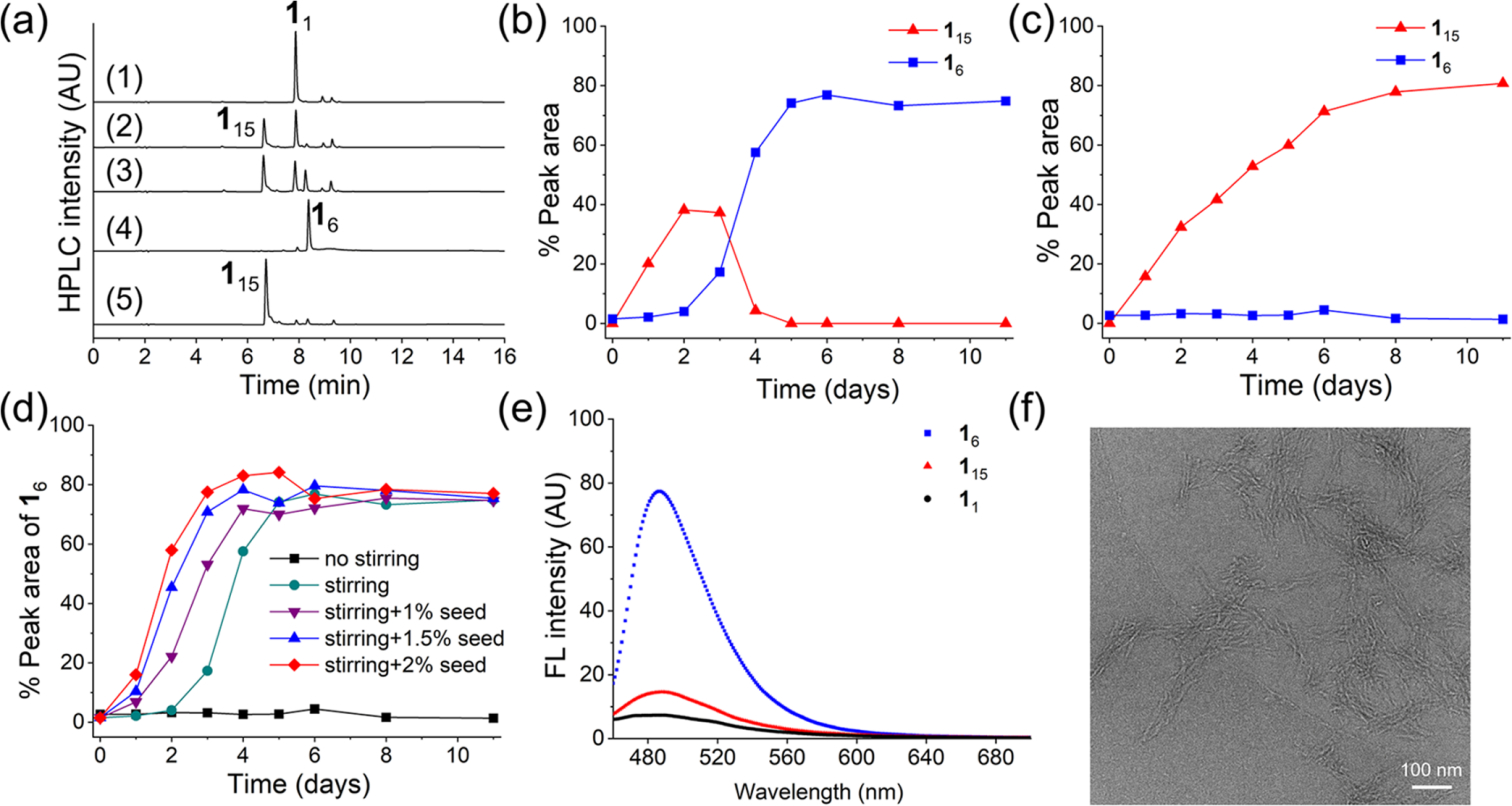
Selective and spontaneous formation of either a self-replicator or a foldamer depending on mechanical agitation. (a) Ultra-high-performance liquid chromatography (UHPLC) of the dynamic combinatorial libraries (DCLs) made from 1.0 mM **1** under magnetic stirring (600 rpm) at day 0 (trace 1), day 2 (trace 2), day 3 (trace 3), and day 8 (trace 4) in 50 mM borate buffer, pH 8.2, 30 °C. Trace 5 shows the UHPLC chromatogram of the DCL without stirring at day 8. (b) Kinetic profiles for libraries prepared from 1.0 mM of building block **1** at 30 °C in 50 mM borate buffer, pH 8.2 under magnetic stirring (600 rpm) and (c) without stirring. (d) Kinetic profiles for libraries prepared from 1.0 mM of building block **1** in 50 mM borate buffer (pH 8.2) with the addition of different amounts of the preformed **1**_6_ as the seed at day 0. (e) Thioflavin T fluorescence emission spectra of DCLs dominated by **1**_6_, **1**_15_, or **1**_1_. (f) Cryo-transmission electron microscopy (Cryo-TEM) image of the **1**_6_ fibers. Scale bar: 100 nm.

**Scheme 1:**
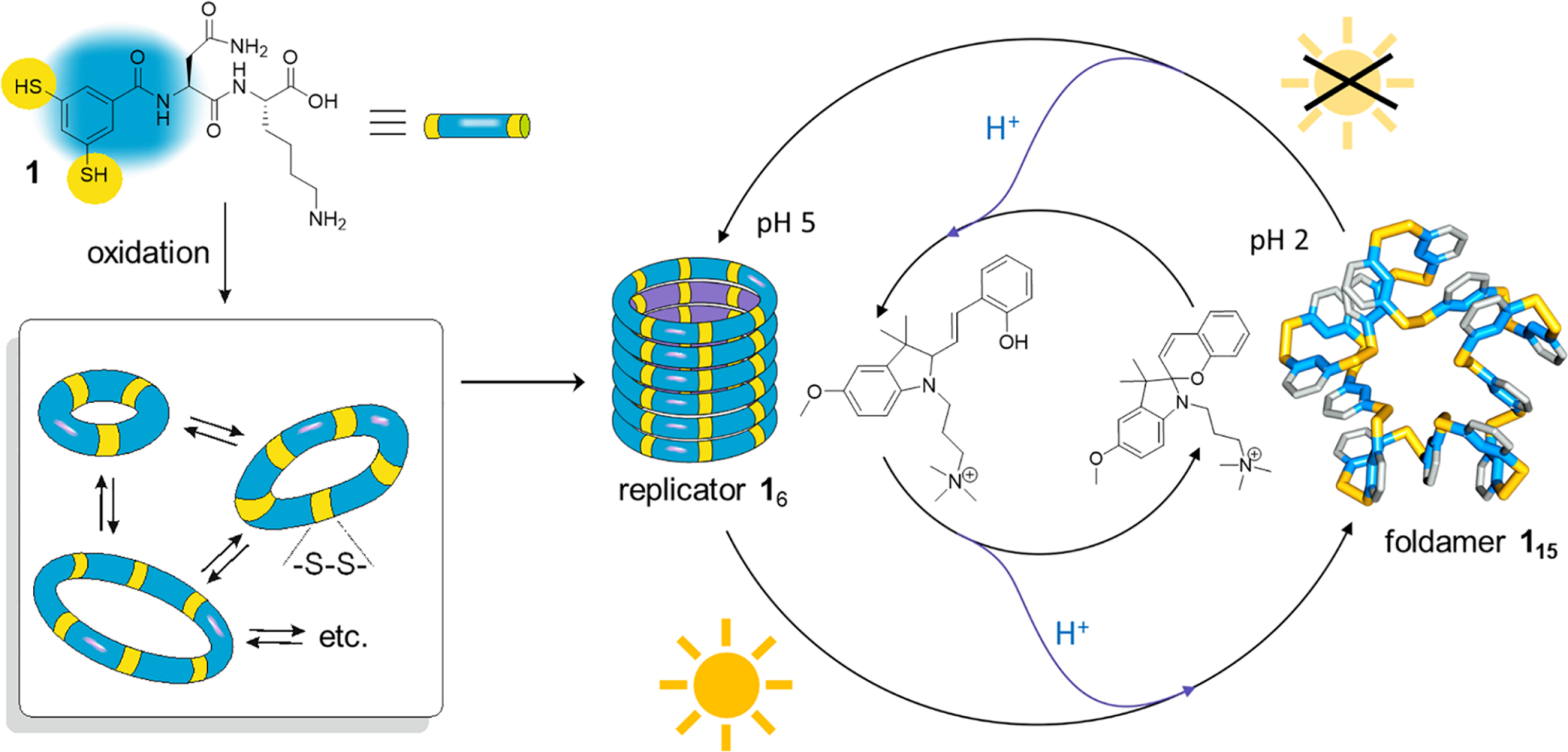
Oxidation by Air of a Solution of Dipeptide-Appended Dithiol Building Block **1** in Water Yields a Dynamic Combinatorial Library (DCL) from Which Foldamer **1**_15_ and Self-Replicator **1**_6_ Can Emerge Photoirradiation releases protons from a photoacid that causes the destabilization of fibrous aggregates of **1**_6_ and its conversion into foldamer **1**_15_. When irradiation is halted, the protons return to the photoacid and the replicator re-forms at the expense of the foldamer.

As part of our exploration of dipeptide-appended dithiol building blocks to access large macrocyclic foldamers,^21,22^ we designed building block **1** (Scheme 1). An asparagine residue was introduced to provide directional interactions (hydrogen bonds) that are important to stabilize specific folded structures. A lysine residue at the C-terminus makes the building block zwitterionic at neutral pH, ensuring sufficient water solubility. Oxidation of building block **1** in borate buffer (pH 8.2) under magnetic stirring (600 rpm) led to the rapid formation of macrocycle **1**_15_, consisting of 15 subunits of **1** (traces 1 and 2 in Figure 1a,b). In the subsequent days, the concentration of **1**_15_ gradually diminished and gave way to hexameric macrocycle **1**_6_, which appears to be the thermodynamic product (Figure 1a(3, 4),b). However, **1**_15_ can be obtained selectively and remained stable for a least 1 month, when the same experiment was conducted without agitation (see trace 5 in Figures 1a,c and S2–S7). Seeding experiments were performed to investigate whether the formation of **1**_6_ is an autocatalytic process. As shown in Figure 1d, when a fresh library was seeded with preformed **1**_6_ its rate of formation increased compared to the samples, where no seed was added. The effect was more pronounced when the amount of seed increased from 1 to 2%. These results confirm that **1**_6_ is a self-replicator. The observation that stirring promotes the formation of **1**_6_ is consistent with growth through a fiber elongation–fragmentation mechanism, as proposed previously for similar self-replicating molecules.^35^ In the absence of stirring, fibers do not readily fragment, and the growth of **1**_6_ is hampered, allowing **1**_15_ to persist. Note that stirring speed influences the rate of replicator formation,^35^ but that harsher methods like sonication have been found to degrade the material due to overoxidation of the disulfide linkages to sulfinic and sulfonic acids.

We conducted thioflavin T assays (Figure 1e), which showed enhanced fluorescence at 487 nm in the presence of **1**_6_ but not for **1**_15_ and **1**_1_, indicative of β-sheet-like structures in the assemblies of **1**_6_. Cryo-TEM images of a sample dominated by **1**_6_ showed bundles of fibers that, individually, have a diameter of 3.5 nm (Figure 1f). This dimension is in agreement with the diameter of a single hexamer macrocycle, with peptide chains extending radially from the dimercaptobenzene core.

### Characterization of the 15mer Foldamer

**Figure 2:**
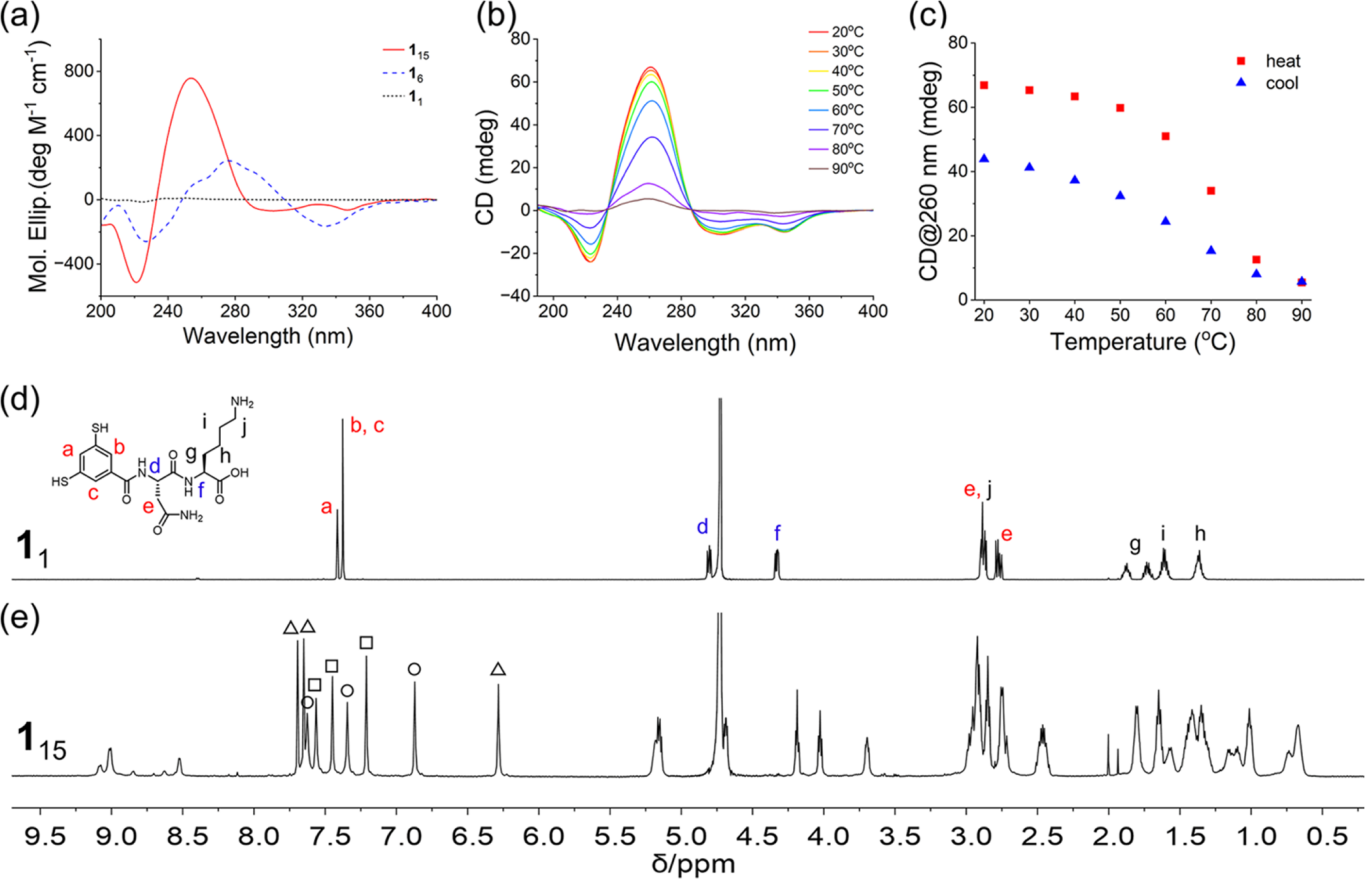
Characterization of **1**_15_ by CD and NMR. (a) CD spectra of **1**_15_, **1**_6_, and **1**_1_. (b) Variable-temperature CD spectra of **1**_15_ from 20 to 90 °C. (c) Changes in ellipticity at 260 nm of **1**_15_ upon a heat–cool cycle. ^1^H NMR spectra of (d) **1**_1_ and (e) **1**_15_ in D_2_O (600 MHz). The peaks marked with triangles, circles, and squares belong to the aromatic protons of three distinct phenyl rings, respectively (inferred from the total correlation spectroscopy (TOCSY) in Figure S10). The exact assignment to a-, b-, and c-type protons was not performed.

Macrocycle **1**_15_ was purified by HPLC and characterized with circular dichroism (CD), ^1^H NMR spectroscopy, and X-ray crystallography. While the solution of building block **1** showed only weak CD signals, **1**_15_ gave an intense positive band at 260 nm, indicating that the aromatic core experiences a chiral environment (Figure 2a). In comparison, **1**_6_ shows a positive peak at 278 nm. The bathochromic shift of the band with respect to **1**_15_ was assigned to π-stacked phenyl rings. A shoulder at 260 nm was also detected. In addition, the maximum at around 210 nm and the minimum at around 230 nm suggest the formation of β-sheet structures in the assemblies of **1**_6_. Temperature-dependent CD experiments show that the signal of **1**_15_ at 260 nm decreases by 92% after the temperature is increased from 20 to 90 °C (Figure 2b). The signal recovered only 66% of its original value when the temperature was decreased back to 20 °C (Figures 2c and S8a). HPLC analysis of the sample showed that **1**_15_ had decomposed to smaller macrocycles after the heat–cool cycle (Figure S8b). For the **1**_6_ sample (Figure S9a), the CD signal at 278 nm decreased by 53% after elevating the temperature from 20 to 90 °C but recovered 84% after cooling. HPLC analysis of the sample indicated that **1**_6_ was partially converted to larger macrocycles upon thermal cycling (Figure S9b).

The solution-phase ^1^H NMR spectra (D_2_O, 298 K) of **1**_1_ and **1**_15_ are shown in Figure 2d,e. Foldamer **1**_15_ shows three sets of signals for the aromatic protons (from 6.3 to 7.8 ppm), which suggests that **1**_15_ has an average *C*_5_ symmetry. Furthermore, the pairs of aromatic protons *ortho* to each benzamide function (labeled b and c in Figure 2d) appear as distinct signals in the spectrum of **1**_15_, indicating a loss of symmetry for each building block (also see TOCSY spectrum in Figure S10). The large upfield chemical shifts of two phenyl protons to 6.9 and 6.3 ppm indicate that some of these protons are adjacent to the face of an aromatic ring. The sharp and well-defined NMR spectrum suggests that **1**_15_ adopts a highly ordered structure in solution.

**Figure 3:**
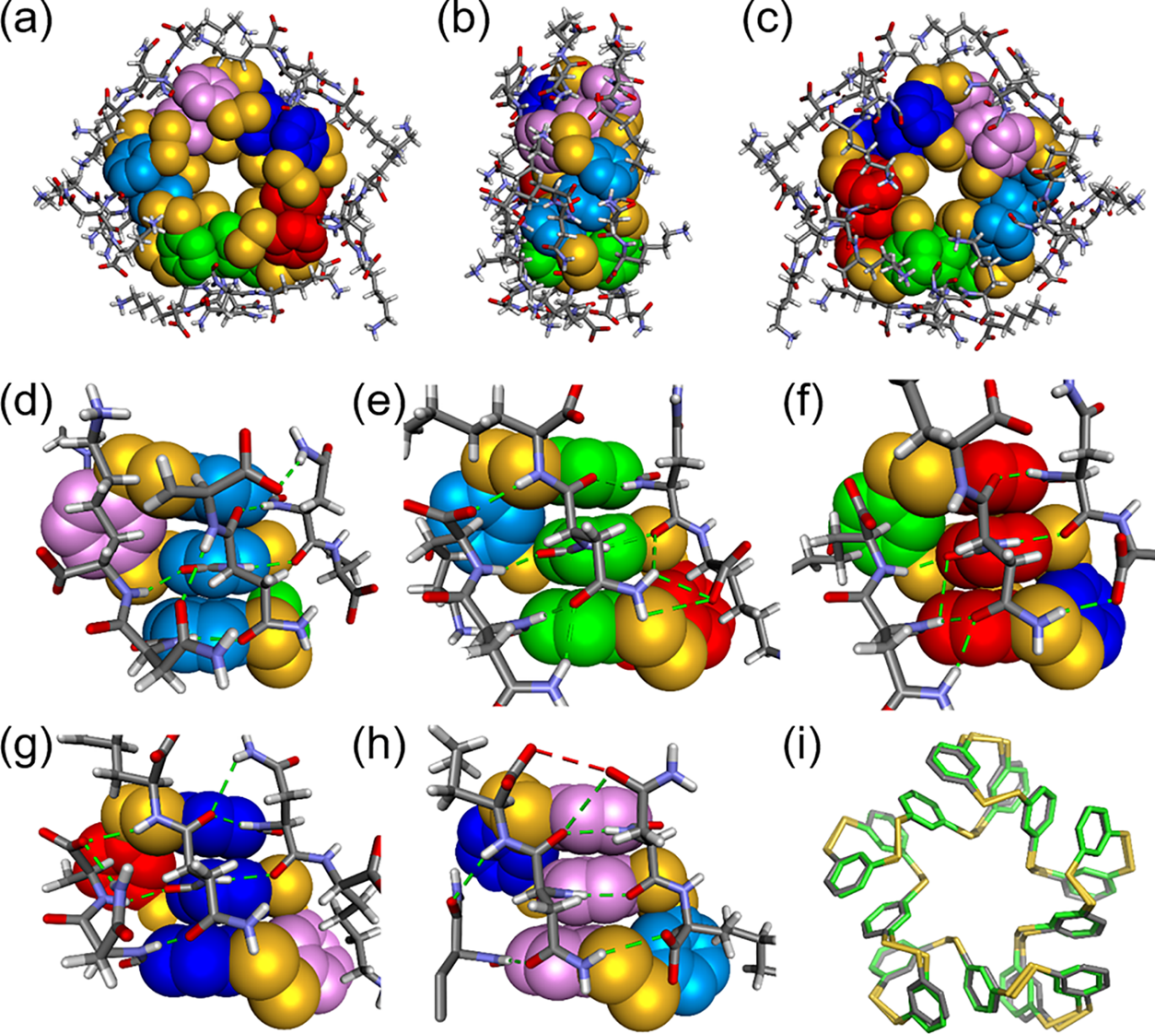
Crystal structure of foldamer **1_15_**. (a) Top, (b) side, and (c) bottom views of the color-coded space-filling representation of the hydrophobic dimercaptobenzene core and tube representation of the side chains of L-**1**_**15**_ from the crystal structure of L/D-**1**_**15**_. (d–h) Front view of the five stacks of the three core benzene rings, highlighting the arrays of hydrogen bonds (green dashes), (h) also highlighting the –CO_2_H···O=C contact (red dashes). (i) Tube representation of the hydrophobic core of L-**1**_**15**_ (gray) aligned sterically to the hydrophobic core of an analogous pentagonal structure (green) formed from the assembly of 15 units of building block bearing aspartic acid and an adenine residue instead of the dipeptide in **1**.^21^

The crystal structure of **1**_15_ shows that the 15 dimercaptobenzene moieties collapse into a dense hydrophobic core exposing their hydrophilic appendages at the surface, resembling the folding of proteins (Figures 3, S12, and S13). The structure is very well-defined with 14 out of 15 lysine residues being visible in the electron-density map. Five identical stacks of three phenyl rings are found in the core, consistent with the 5-fold symmetry observed in the NMR spectrum. Overlay of this core structure with another previously discovered 15mer fold^21^ indicates that they are very similar (Figure 3i). The similarity in the folding of the hydrophobic core is remarkable, given that the previously reported 15mer is made from a building block that has a substantially different structure, featuring an aspartic acid and an adenine residue instead of the dipeptide of **1**. Most of the butyl chains of the lysine residues of **1**_15_ lay down on the aromatic core and establish hydrophobic contacts with the faces of the stacks of the three benzene rings. In the previously described 15mer, adenine rings were found to stack at these positions. Extensive hydrogen bonding occurs between dipeptide residues within each of the five subsets of **1**_15_ but not between them (Figure 3d–h). Apart from the amide bonds in the peptide backbone, the side-chain amides from asparagine together with side-chain amines and C-terminal carboxylate groups from lysine are shown to play an important role in these hydrogen-bonding interactions. Note that the hydrogen-bonding patterns differ from subset to subset in the crystal structure (Figure 3d–h). These different patterns must exchange rapidly on the NMR time scale to give rise to the observed average 5-fold symmetry.

### Foldamer **1**_15_ and Replicator **1**_6_ Interconvert upon Changing the pH

**Figure 4:**
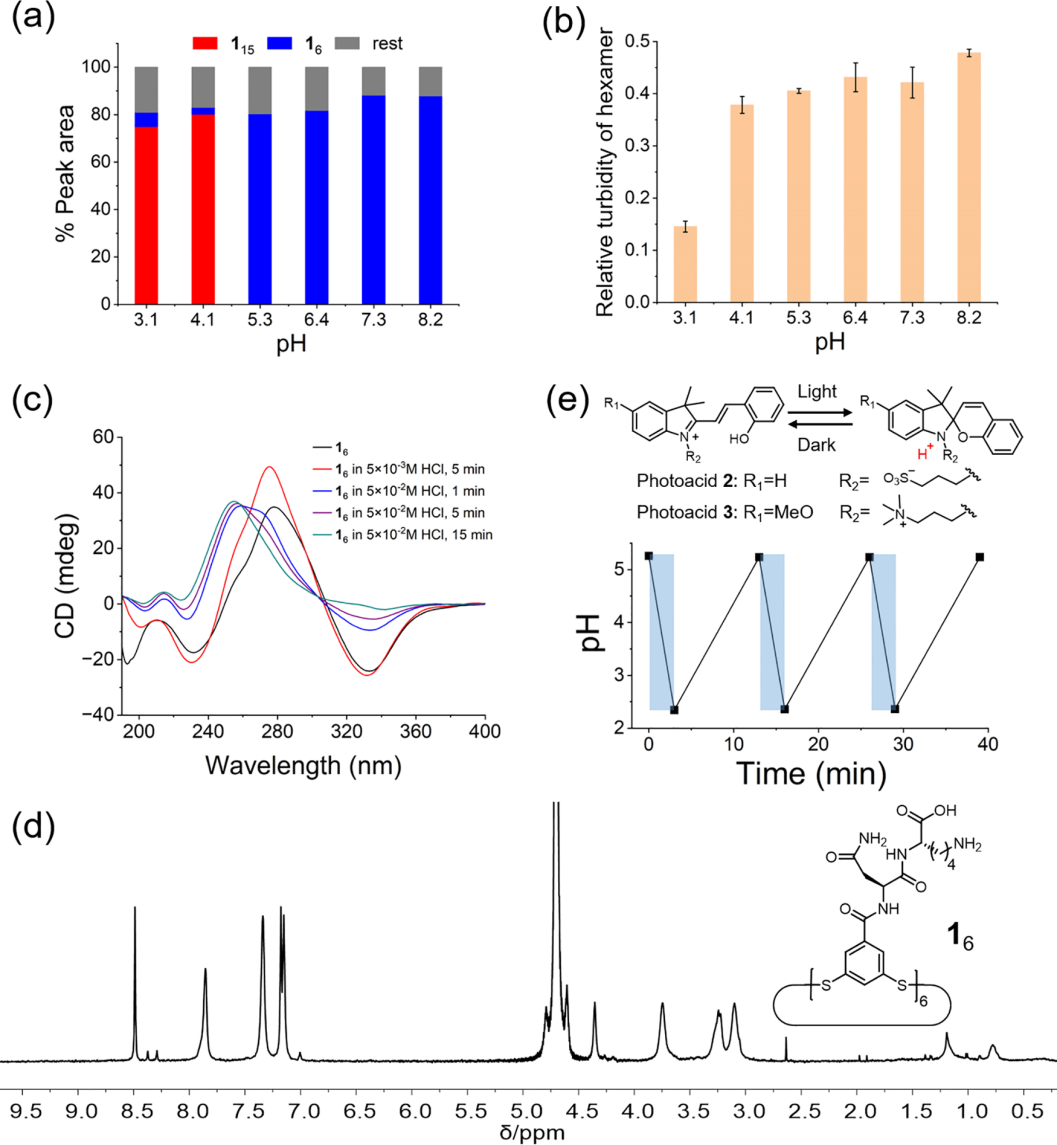
Effect of pH on the formation of foldamer **1**_**15**_ and replicator **1**_**6**_. (a) UHPLC peak area ratios for libraries prepared from 1.0 mM building block **1** in citrate-phosphate buffer of different pH values after 8 days of magnetic stirring (600 rpm). (b) Relative turbidity of a sample of preformed fibers of **1**_6_ (0.40 mM in building block **1**) in citrate-phosphate buffer solutions of different pH values. Relative turbidity was calculated as (*A* – *A*_0_)/*A*_0_, where *A* is the absorbance of **1**_6_ sample at 650 nm, and *A*_0_ is the absorbance of the corresponding buffer at 650 nm. (c) CD spectra of **1**_6_ in water after adding different amounts of HCl. (d) ^1^H NMR spectrum of **1**_6_ in D_2_O containing 0.5 M HCl (600 MHz). (e) Chemical structures of two photoacids and the pH change of a photoacid **3** (MCH) solution upon alternating between periods of irradiation and no irradiation. For each cycle, a solution of MCH at a concentration of 5.0 mM was irradiated with an light-emitting diode (LED) (450 nm) for 3 min and kept in the dark for 10 min. Blue shading indicates the periods of irradiation.

Further investigation revealed that lowering the pH favors the formation of the 15mer foldamer and inhibits the formation of the competing replicator, even under magnetic stirring. As shown in Figures 4a and S14, when the pH was high (i.e., pH 8.2, 7.3, 6.4, and 5.3), replicator **1**_6_ dominated the library, while at lower pH (i.e., pH 4.1 and 3.1), **1**_15_ was formed selectively. Some rationale for the stability of **1**_15_ at low pH was found in its crystal structure (Figure 3h).

The crystallization of **1**_15_ was performed at a pH above 8.0. C-terminal functions thus exist as carboxylates. Only two of them were found to be involved in “salt-bridges”, i.e., charge-reinforced hydrogen bonds, with lysine residues (*d*_N···O_ < 3.5 Å) susceptible to be altered upon protonation of the carboxylate. The others are either simply exposed to the water (five) or hydrogen-bonded to peptidic or Asn amide protons, that is, less susceptible to alter the overall fold stability upon carboxylate protonation. In some cases, carboxylate protonation may even provide an additional hydrogen bond involving the acid proton as a donor.

In contrast, even though the structure of replicator **1**_6_ is unknown, its assembly is hampered upon acidifying the medium: both the assembly of **1**_6_ into fibers and associations between these fibers are disrupted. Evidence comes from UV–vis, CD, TEM, and NMR data. The aggregation of preformed **1**_6_ fibers in buffer solutions of different pH values was analyzed by UV–vis spectroscopy and turbidity measurement (absorbance at 650 nm; Figure 4b). As the pH of the solution was changed from high (8.2, 7.3, 6.4, 5.3, 4.1) to low (3.1); the turbidity of the **1**_6_ sample decreased significantly, indicating that the **1**_6_ fibers are less aggregated at lower pH. The effect of pH on the assembly of **1**_6_ was investigated by CD and TEM. As shown in Figure 4c, when different amounts of HCl were added to a sample of **1**_6_ fibers, the peak intensity at 278 and 330 nm, which are characteristic for fibers of stacks of **1**_6_, first increased a little but then gradually diminished, with the emergence of a new peak at around 260 nm. HPLC analysis (Figure S15b) showed that the **1**_6_ rings nevertheless remained intact, suggesting that the shift of the CD signal can be attributed to a change in the mode of assembly of **1**_6_. Variable-temperature CD data of a sample of **1**_6_ in the presence of 50 mM HCl (Figure S15a) exhibit a decrease and complete recovery of the CD signal at 260 nm during a heat–cool cycle (20–80–20 °C). HPLC analysis of the same sample confirmed that **1**_6_ remained intact during this treatment (Figure S15b). Negative stain TEM images showed that at lower pH, fibers disassembled into smaller ones (Figure S16). Finally, ^1^H NMR spectra of a sample of **1**_6_ in the presence of 0.5 M HCl (Figure 4d) exhibited sharp peaks, which indicated the presence of unstacked hexamer rings. In contrast, no signals were detected in NMR spectra of samples of fibrous **1**_6_ at close-to-neutral pH. Different from ^1^H NMR spectra of building blocks **1** and **1**_15_ (Figure 2d,e), downfield-shifted signals (i.e., 8.49 and 7.86 ppm) were detected for **1**_6_ (Figure 4d). HPLC analysis confirmed that **1**_6_ in a 0.5 M HCl solution remained stable for 11 days (Figure S17).

We speculate that when the pH is lower than the p*K*_a_ of carboxylic acid (typically in the range of 4.2–4.7), building block **1** will attain a net positive charge, resulting in electrostatic repulsion that will destabilize the fibers of **1**_6_. When there is sufficient thiol available for disulfide exchange, such destabilization will then result in a re-equilibration in favor of foldamer **1**_15_.

### Light-Controlled Interconversion between Replicator and Foldamer

The pH responsiveness of the system provides an opportunity for the dynamic switching between the 15mer foldamer and hexamer self-replicator. At higher pH, the thermodynamically more stable hexamer fiber dominates the library at the expense of the other species. At lower pH, hexamer fibers disassemble and the formation of the 15mer foldamer becomes favored. We reasoned that it should be possible to drive the interconversion between the two oligomers by the photoirradiation of an appropriately chosen photoacid. We first tested photoacid **2** (Figure 4e).^36^ However, the poor hydrolytic stability^37^ of this compound, together with its limited solubility, restricted its use. We then focused on the recently developed photoacid **3**(38,39) (Figure 4e). This compound has a methoxy group on the indolinium ring, which was reported to improve hydrolytic stability. Furthermore, its positively charged alkyl ammonium side chain greatly enhances its water solubility, which enables a wider pH switching window. Upon light irradiation, the open-ring protonated merocyanine (MCH) switches to the closed spiropyran form (SP) with the release of a proton. As shown in Figure S19, the UV-vis spectrum of MCH before irradiation exhibited a characteristic adsorption peak at 440 nm, which decreased upon 10 s of irradiation, while the peak at 242 nm increased, which was attributed to the spiropyran form. The pH of the MCH aqueous solution (5.0 mM) changed from 5.2 to 2.4 upon irradiation. When irradiation is stopped, the absorbance at 440 nm restores in 5 min and the pH returns to the original value (Figure 4e). Such a pH switching range fits well with the pH change needed for interconverting replicator and foldamer.

**Figure 5:**
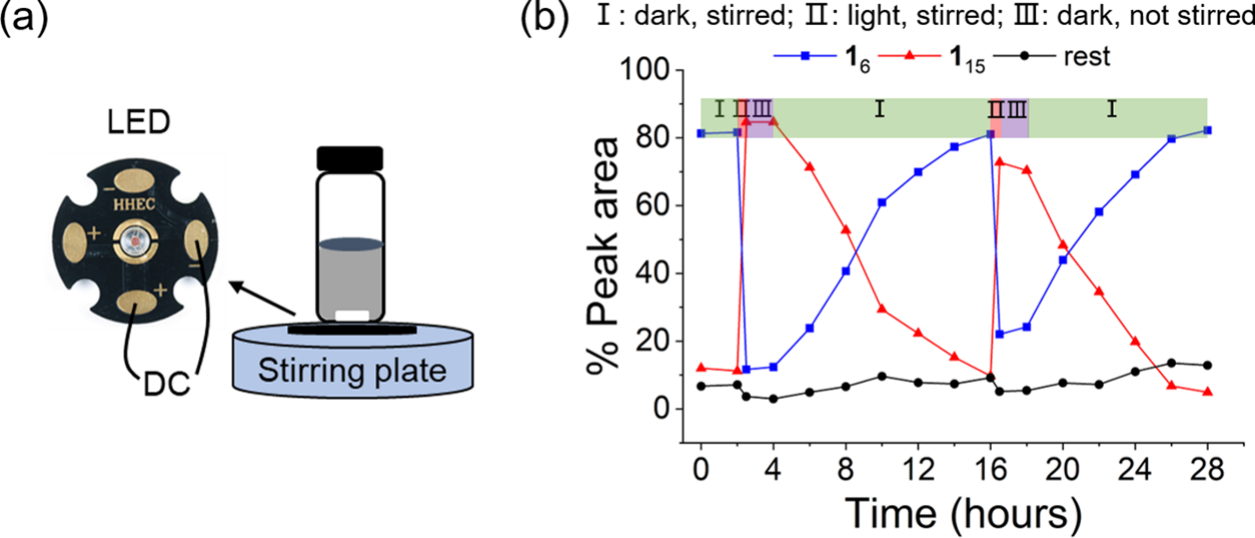
Light-controlled interconversion between the replicator and foldamer. (a) Experimental setup in which a DCL in the presence of photoacid **3** was irradiated by an LED and magnetically stirred at 600 rpm. A nitrogen atmosphere was used to prevent the rapid oxidation of free thiols in the sample. (b) Kinetic profile of the dynamic library made from **1** in the presence of photoacid **3** (5.0 mM) during two light irradiation–darkness cycles. The experiment was started from premade fibers of **1**_6_ to which 0.10 equiv of **1** was added to mediate disulfide exchange. The initial pH of the solution was 5.2.

We then probed the photomediated interconversion between these two compounds using the setup shown in Figure 5a. Reaction monitoring was performed by UHPLC. The strong absorbance of photoacid **3** did not allow for simultaneous monitoring by CD because the transmittance was too low. We started from a mixture of **1**_6_ fibers and MCH that was stable as long as it was not irradiated (Figures 5b and S20). When the light was switched on (at *t* = 2 h) for 30 min, the concentration of foldamer **1**_15_ increased rapidly and dominated the library, while replicator **1**_6_ was consumed. We then turned off the light and the stirring, whereafter the composition of the library barely changed, as the system became trapped in a metastable state (from 2.5 to 4 h). When stirring was commenced, **1**_6_ grew to dominate the library in the course of 12 h. A second round of switching between replicators and foldamers was also realized by another light–dark cycle. Thus, through the use of light as a source of energy, the system can be taken out of its equilibrium state, where it is dominated by replicator **1**_6_ and form a dissipative stationary state dominated by foldamer **1**_15_ that could be maintained as long as it was irradiated (Scheme 1).

## Conclusions

We have demonstrated the spontaneous formation of a foldamer, consisting of 15 building block units, or a self-replicator, consisting of 6 building block units, from a dynamic combinatorial library upon oligomerization of building block **1**, as well as their interconversion triggered by a change in agitation regime or pH.

In general, in dynamic combinatorial libraries, smaller compounds, like dimers, trimers, and tetramers, tend to be the thermodynamically more stable species, as producing a large number of small library members is entropically favorable over producing a smaller number of larger library members. However, the formation of noncovalent interactions (enthalpy-driven, if we disregard (de)solvation effects) by specific library members can overcome this inherent bias. In the present systems, at moderately elevated pH, these noncovalent interactions form either intermolecularly (driving the self-assembly of **1**_6_ into a supramolecular polymer) or intramolecularly (driving the folding of **1**_15_). At this pH, the nucleation of the fiber is accompanied by a large kinetic barrier (most likely the result of the large entropic penalty associated with the conformational rearrangement of the interacting molecules), while, perhaps counterintuitively, the foldamer forms more readily. So, the pathway to foldamer formation is associated with a lower kinetic barrier than that leading to replicator nucleation. However, once an autocatalytic path (promoted by fiber fragmentation upon agitation) opens up, it outcompetes foldamer formation and even converts the already formed foldamers into thermodynamically more stable replicators.

Mechanical agitation at close-to-neutral pH enables the conversion of foldamer **1**_15_ to thermodynamically more stable self-replicator **1**_6_, while lowering the pH causes foldamer **1**_15_ to become the dominant species. Using a photoacid, the interconversion between the replicator and foldamer was achieved by switching photoirradiation on or off. Here, the energy of light was used to move the system away from the equilibrium state, where the replicator dominates, to produce a high steady-state concentration of foldamer that was maintained as long as irradiation continued. This light-mediated switching between replicator and foldamer states complements other work, published in this issue, in which homeostatically populating the foldamer state was based on chemical fueling, requiring the continuous supply of building block material. Coupling between replication and foldamer formation creates a system where both species can be accessed and constitutes a primitive way of coupling a genotype (the replicator) to a phenotype (the foldamer). The challenge is now to endow the foldamer with a function (for example, catalytic activity) that would benefit the system, which would allow for the selection of such genotype–phenotype coupling in the course of the Darwinian evolution.

## Supporting Information Available

General procedures, building block synthesis, library preparation, UHPLC-MS analysis, thioflavin T fluorescence, (cryo-)TEM, (variable temperature) circular dichroism, turbidity, NMR and X-ray crystallography data, pH measurements, and light-triggered switching between self-replicator and foldamers (PDF)
