# Supplementary material for: Light-Mediated Interconversion between a Foldamer and a Self-Replicator
Source: J Am Chem Soc. 2024 Nov 26;146(49):33395–402. doi: 10.1021/jacs.4c09114 (PMC11638899; doi:10.1021/jacs.4c09114)
Supplement: Supplementary file 1 — ja4c09114_si_001.pdf [file ja4c09114_si_001.pdf]

Supporting Information

Light-Mediated Interconversion Between a Foldamer and a Self-Replicator

Yulong Jin[1,4*], Pradeep K. Mandal[2], Juntian Wu[3], Armin Kiani[3], Rui Zhao[1], Ivan Huc[2,*] and Sijbren Otto[3,*]

[1] Beijing National Laboratory for Molecular Sciences, CAS Key Laboratory of Analytical Chemistry for Living Biosystems, Institute of Chemistry, Chinese Academy of Sciences, Beijing, China

[2] Department of Pharmacy, Ludwig-Maximilians-Universität München, Munich, Germany

[3] Centre for Systems Chemistry, Stratingh Institute, University of Groningen, Groningen, the Netherlands

[4] State Key Laboratory of Chemical Resource Engineering, Beijing Advanced Innovation Center for Soft Matter Science and Engineering, College of Chemistry, Beijing University of Chemical Technology, Beijing, China

Table of Contents

1. Experimental section 3

   1.1 General procedures 3

   1.2 Peptide synthesis 4

   1.3 Library preparation 4

   1.4 UHPLC analysis 5

   1.5 UHPLC-MS analysis 5

   1.6 Thioflavin T (ThT) fluorescence assay 6

   1.7 Cryo-TEM measurements 6

   1.8 CD spectroscopy 7

   1.9 Turbidity measurements 7

   1.10 Negative stain Electron Microscopy (NS-EM) 7

   1.11 Light irradiation and pH measurements 8

   1.12 Light-mediated switching between self-replicator and foldamer 8

   1.13 X-ray crystallography of $\mathbf{1}_{15}$ 8

2. Supporting tables and figures 11

3. References 32

1. Experimental section

1.1 General procedures

Fmoc-amino-acid-Wang resins, Fmoc-Asn(Trt)-OH, and 2-(1H-benzotriazole-1-yl)-1,1,3,3-tetramethyluronium hexafluorophosphate (HBTU) were purchased from GL Biochem (Shanghai, China). 1-Hydroxybenzotriazole hydrate (HOBT·H_2O) was obtained from Siam (Chicago, USA). Dimethylformamide (DMF) and acetonitrile (HPLC grade) were purchased from Fisher Scientific (Hampton, USA). Dichloromethane (DCM), piperidine, 4-methylmorpholine (NMM), diethyl ether, disodium hydrogen phosphate dodecahydrate ($Na_2HPO_4 \cdot 12H_2O$) and citric acid were all purchased from Beijing Chemical Works (Beijing, China). 1,2-Ethanedithiol (EDT), triisoproyl silane (TIS), trifluoroacetic acid (TFA) were obtained from Sigma-Aldrich. Ultrapure water from a Milli Q water purification system (Burlington, USA) was used throughout. Other materials used for synthesis were commercially available and used as received. NMR spectra were recorded on Bruker Avance 600 MHz spectrometers. HRMS spectra were recorded on an Orbitrap Fusion Lumos Tribrid Mass Spectrometer. UV-Vis adsorption spectra were recorded on a Shimadzu UV-1800 spectrometer (Kyoto, Japan).

Photoacid **3** (MCH) was synthesized following a literature procedure.[1] ^{1}H-NMR (700 MHz, DMSO-d_6, 298K, shown in Figure S19). δ 7.17 (dd, J = 7.5, 1.7 Hz, 1H), 7.10 (td, J = 7.7, 1.7 Hz, 1H), 7.00 (d, J = 10.2 Hz, 1H), 6.84 (td, J = 7.4, 1.1 Hz, 1H), 6.79 (d, J = 2.6 Hz, 1H), 6.70 – 6.65 (m, 2H), 6.56 (d, J = 8.4 Hz, 1H), 5.82 (d, J = 10.2 Hz, 1H), 3.26 (td, J = 12.4, 4.8 Hz, 1H), 3.16 (dt, J = 14.7, 7.4 Hz, 1H), 3.05 (ddd, J = 14.7, 7.0, 5.5 Hz, 1H), 3.00 (s, 8H), 2.01 (dq, J = 12.5, 5.4 Hz, 1H), 1.90 (dt, J = 12.5, 6.0 Hz, 1H), 1.19 (s, 3H), 1.12 (s, 3H). MALDI-FTICR-MS, monoisotopic m/z calculated for $C_{25}H_{33}N_2O_2^+$ $[M]^+$: 393.2537, observed: 393.2537.

1.2 Peptide synthesis

The synthesis of the building block **1** was performed using Fmoc solid-phase peptide synthesis on Wang resin. Amino acids were introduced as Fmoc-Lys(Boc)-OH, and Fmoc-Asn(Trt)-OH. Fmoc deprotection steps were carried out with 20% piperidine in DMF (2×5 min). In the coupling of subsequent amino acid or 3,5-bis(tritylthio)benzoic acid, 3-fold Fmoc-protected amino acids or 3,5-bis(tritylthio)benzoic acid in the presence of 3-fold HBTU, 3-fold HOBT and 8-fold NMM were used. Deprotection from the resin and removal of the protecting groups from the side chains of the amino acids were performed using a cocktail of 94% TFA, 2.5% EDT, 2.5% water and 1% TIS for 2 h. The solution was then filtered and the filtrate was vacuum-dried by rotary evaporation and the residue was treated with cold diethyl ether. The white precipitate was collected by centrifugation and washed with cold diethyl ether three times. The HPLC purification of building block **1** and the oligomerized cyclic compounds was performed on a Shimadzu LC-20AR HPLC system with an Ultimate XB-C18 column (Welch, 21.2×250 mm, 5 μm). HPLC purified products all have a purity higher than 95%. MALDI-FTICR-MS, monoisotopic m/z calculated for $C_{17}H_{25}N_4O_5S_2^+$ $[M+H]^+$:429.1261, observed: 429.1263.

1.3 Library preparation

Borate buffer (50 mM in $Na_2B_4O_7$, pH 8.2) was prepared by dissolving $Na_2B_4O_7 \cdot 10H_2O$ (9.5 g, 25 mmol) in 480 mL ultrapure water. The pH was then adjusted to 8.2 using concentrated HCl. Additional water was added to set the final volume of the solution to 500 mL. Citrate-phosphate buffers with different pH from 3.1 to 8.2 were prepared by mixing different volumes of Na_2HPO_4 solution (20 mM) and citrate solution (10 mM). Building blocks (final concentration of 1.0 mM) were dissolved in 50 mM borate buffer (pH 8.2) or citrate-phosphate buffer. All the

libraries were set up in HPLC vials (12 × 32 mm) with a Teflon-coated screw cap. HPLC vials were equipped with a cylindrical stirrer bar (2 × 5 mm, Teflon coated) and stirred at 600 rpm using an IKA RCT basic hot plate stirrer. All experiments were performed at 30 °C unless otherwise specified.

1.4 UHPLC analysis

UHPLC analyses were performed on a Shimadzu LC-40D XR UHPLC system. The separation systems were all equipped with a photodiode array detector at a detection wavelength of 254 nm. Samples were analyzed on a HALO peptide ES-C18 column (160 Å, 2 µm, 2.1 × 150 mm), using water (eluent A) and acetonitrile (eluent B), which contained 0.1 v/v trifluoroacetic acid as the modifier. A flow rate of 0.2 mL min^{-1} and a column oven temperature of 30 °C were applied. Gradient: 0-12-12.2-16min, 5%-60%-5%-5% B. Samples were prepared by diluting 10 µL of the library with 40 µL of ultrapure water. HPLC injection volume was 5 µL.

1.5 UHPLC-MS analysis

UHPLC-MS analyses were performed on a Thermo Scientific UltiMate 3000 UHPLC system coupled to a Thermo Scientific Fusion Lumos Orbitrap mass spectrometer. Samples were analyzed on an Acquity UPLC Protein BEH C4 column (300 Å, 1.7 µm, 2.1 × 150 mm), using water (eluent A) and acetonitrile (eluent B), which contained 0.1 v/v formic acid as the modifier. A flow rate of 0.2 mL min^{-1} and a column oven temperature of 35 °C were applied. Gradient: 0-3-20-22 min, 5%-5%-70%-70% B. Samples were prepared by diluting 5 µL of the library with 30 µL of double distilled water. UHPLC injection volume was 3 µL. The mass spectrometer was operated in a positive electrospray ionization mode with the ionization parameters: capillary

voltage, 3 kV; sheath gas (Arb), 35; aux gas (Arb), 15; sweep gas (Arb), 2; ion transfer tube temperature, 280 °C; vaporizer temperature, 150 °C; orbitrap resolution, 120000; scan range (m/z), 200-2000; RF lens (%), 30; AGC target, $4.0e^5$; maximum injection time (ms), 50; microscan, 1; data type, profile.

1.6 Thioflavin T (ThT) fluorescence assay

Fluorescence measurements were performed on a Hitachi F-4600 fluorescence spectrophotometer (Tokyo, Japan) using quartz cuvettes with 1 cm path length. For the ThT measurements, a freshly prepared solution of thioflavin T (dissolved to 2.2 mM in 50 mM borate buffer at pH 8.2 and filtered through a 0.2 μm syringe filter) was diluted to 22 μM with the same buffer. An aliquot of 400 μL of this diluted solution was transferred to the cuvette, followed by the addition of 100 μL of sample. The final concentration of sample was 20 μM (in building block **1**). Spectra were recorded after an incubation time of 2 minutes. The excitation wavelength was set at 440 nm and spectra were recorded in the range of 460-700 nm with a slit width of 5 nm.

1.7 Cryo-TEM measurements

The samples for the Cryo-TEM observation were prepared with a Vitrobot Mark IV (ThermoFisher Scientific). Before the preparation of samples, we put the grid in the Vitrobot Mark IV at room temperature. An aliquot of 3 μL of the hexamer sample solution was dripped onto a holey carbon film on a copper grid (GIG) and blotted with a piece of filter paper to obtain a thin liquid film on the grid. Then the grid was quickly plunged into liquid ethane. The vitrified samples were then transferred to a 626 cryogenic sample holder (Gatan) and examined with a

Cryo-TEM (Themis 300, ThermoFisher Scientific) at 77 K. Micrographs were captured with a Ceta S camera (ThermoFisher Scientific).

1.8 CD spectroscopy

Spectra were recorded on a Jasco J-1700 spectrometer with a Peltier temperature controller. Heat-cool cycles were applied from 20 to 90 °C in steps of 10 °C at a rate of 5 °C min^{-1}. Spectra were obtained as averages of two measurements from 190 to 400 nm with a scanning speed of 500 nm min^{-1} and a bandwidth of 1 nm. A quartz cuvette with a 1 cm path length was used for the measurements. The concentration of all samples was 0.10 mM in building block unless otherwise specified.

1.9 Turbidity measurements

Turbidity measurements were performed on a microplate reader (Molecular Devices SpectraMax M2e). An aliquot of 20 μL of $\mathbf{1}_6$ sample (2.0 mM in building block **1**) was mixed with 80 μL of citrate-phosphate buffer and added into a 96-well plate. We use 650 nm as the wavelength for the turbidity measurements, and all measurements were performed at room temperature (23.0 ± 0.5 °C). Experiments were carried out in triplicate. Wells with the same volume of buffers were used as blanks.

1.10 Negative stain Electron Microscopy (NS-EM)

A small drop (5 μL) of the samples was deposited on a copper grid covered with a thin carbon film (Agar Scientific). After one minute, the grid was blotted on filter paper. The sample was subsequently stained twice with uranyl acetate by depositing 5 μL of a saturated uranyl acetate

solution on the grid and blotting it on filter paper after incubation for 20 seconds. Samples were recorded on a Philips CM12 transmission electron microscope at 120 kV.

1.11 Light irradiation and pH measurements

The light irradiation was performed with a 19 mm light-emitting diode (LED) (article code: BN204762) purchased from Kingbright through a local distributor (OKAPHONE), connected to a 1.2 Ω resistance and a 4.5 V power source using a homemade set-up. The power dissipation is 3 W with a central wavelength of 450 nm, according to the supplier. A heat sink was used beneath the LED for heat dissipation. The pH of the solution was monitored using a Mettler Toledo SevenCompact pH meter with an InLab® Ultra-Micro-ISM sensor. Every pH point was measured twice to confirm that the reading had stabilized.

1.12 Light-mediated switching between self-replicator and foldamer

Into a photoacid **3** aqueous solution (5.0 mM, 1.0 mL), the preformed $\mathbf{1}_6$ fibers (0.10 mM in building block **1**) and a small amount of monomer (final concentration: 0.010 mM) were added. An LED plate was used as the light source and placed under the vial. The solution was magnetically stirred at 600 rpm. Nitrogen was used as the protection gas to prevent the rapid oxidation of free thiols in the library. The LED light source was switched on and off at the indicated times and the solution was analyzed directly by HPLC. Only the peaks of DCL members were included for peak area ratio calculation (peak areas of photoacid **3** and its hydrolyzed products were not measured).

1.13 X-ray crystallography of $\mathbf{1}_{15}$

Aqueous solutions of L- and D-$\mathbf{1}_{15}$ were prepared by dissolving the lyophilized powder in pure water to a final concentration of 5.0 mM. Racemic L/D-$\mathbf{1}_{15}$ was prepared by mixing the two enantiopure solutions. Crystallization trials were carried with commercial sparse matrix screens-

JBScreen Basic from Jena Bioscience, using the standard sitting drop vapor diffusion method at 293 K. X-ray quality crystals were obtained after three weeks by the addition of 0.8 μL of L/D-$\mathbf{1}_{15}$ and 0.8 μL of 50% w/v (+/-)-2-methyl-2,4-pentanediol, 100 mM Tris buffer at pH 8.5 and 200 mM ammonium di-hydrogen phosphate in the reservoir. For low temperature diffraction measurement, a crystal was fished using a microloop and plunged into liquid nitrogen. The mother liquor served as cryo-protectant for the crystal.

The X-ray diffraction data was collected at the micro-focus, fixed energy beamline ID23-2[2] in European Synchrotron Radiation Facility (ESRF), Grenoble with a Dectris PILATUS3 X 2M detector. Diffraction data was measured at T = 100 K, λ = 0.8731 Å. The crystals were exposed for 0.02 s and 0.2° oscillation per frame. Diffraction data was processed using the program *autoPROC*[3-7]. The crystal belonged to the space group P-1 with unit cell parameters: a = 27.567 (1) Å, b = 28.289 (5) Å, c = 31.738 (8) Å, α = 72.680 (1)°, β = 76.315 (2)°, γ = 79.988 (8)°; V = 22814 (7) Å^3 and 1 molecule per asymmetric unit (Z = 2, Z' = 1). The structure was solved with the program *SHELXT*[8] and refined by full-matrix least-squares method on F^2 with *SHELXL*-2014[9] within *Olex2.*[10] After each refinement step, visual inspection of the model and the electron-density maps were carried out using *Olex2*[10] and *Coot*[11] using $2F_o - F_c$ and difference Fourier ($F_o - F_c$) maps. The initial structure revealed all main-chain atoms and all asparagine side chains of the $\mathbf{1}_{15}$ macrocycle. Side chains of 14 out of 15 lysine chains were refined with full or partial occupancy. AFIX, DFIX and FLAT instructions were used to improve the geometry of molecules. Restraints on anisotropic displacement parameters were implemented with DELU, SIMU and EADP instructions. All non-H atoms of the backbones were refined with anisotropic displacement parameters. From the difference Fourier map a molecule of (+/-)-2-methyl-2,4-pentanediol (MPD) and a phosphate ion were identified (from the crystallization reagent). After several attempts to model the disordered side chains, the SQUEEZE[12] procedure was used to flatten the electron density map. Very disordered side chains and solvent molecules were removed. Calculated total potential solvent accessible void volume and electron count per cell are 8487.6 Å^3 and 3025 respectively. Hydrogen atoms were placed at idealized positions.

Statistics of data collection and refinement of $\mathbf{1}_{15}$ are described in Table S1. The final cif file of $\mathbf{1}_{15}$ was examined in IUCr's *checkCIF* algorithm. Due to the large volume fractions of disordered solvent molecules, weak diffraction intensity and poor resolution, a number of A- and B- level alerts remain in the *checkCIF* file. These alerts are inherent to the data set and

refinement procedures. They are listed below and were divided into two groups. The first group demonstrates weak quality of the data and refinement statistics when compared to those expected for small molecule structures from highly diffracting crystals. The second group relates to decisions made during refinement and is explained below. Atomic coordinates and structure factors of $\mathbf{1}_{15}$ were deposited in the Cambridge Crystallographic Data Centre (CCDC) with accession code 2209327. The data is available free of charge upon request (www.ccdc.cam.ac.uk/).

CheckCIF validation of **(1)**$_{\mathbf{15}}$:

Group 1 alerts (these illustrate weak quality of data and refinement statistics if compared to small molecule structures from highly diffracting crystals):

THETM01_ALERT_3_A The value of sine(theta_max)/wavelength is less than 0.550
    Calculated sin(theta_max)/wavelength = 0.4348
PLAT029_ALERT_3_A _diffrn_measured_fraction_theta_full value Low . 0.908
PLAT084_ALERT_3_B High wR2 Value (i.e. > 0.25) .... 0.43 Report
PLAT241_ALERT_2_B High 'MainMol' Ueq as Compared to Neighbors of Check
PLAT242_ALERT_2_B Low 'MainMol' Ueq as Compared to Neighbors of Check
PLAT340_ALERT_3_B Low Bond Precision on C-C Bonds 0.02382 Ang.
PLAT414_ALERT_2_B Short Intra D-H..H-X H04T ..H07P 1.81 Ang.
PLAT430_ALERT_2_A Short Inter D...A Contact O03F ..O08A 2.46 Ang.

Group 2 alert (is connected with decision made during refinement and explained below):

PLAT306_ALERT_2_B Isolated Oxygen Atom (H-atoms Missing ?) Check
    Dummy O atom was introduced into refinement.

2. Supporting tables and figures

Table S1. Crystallographic data and refinement details for $\mathbf{1}_{15}$.

Identification code	L/D-$\mathbf{1}_{15}$
Empirical formula	$C_{253.5} H_{322.5} N_{57.5} O_{78.5} P_{0.5} S_{30}$
Formula weight	6408.46
Temperature	100.15 K
Wavelength	0.8731 Å
Crystal system	Triclinic
Space group	*P*-1
Unit cell dimensions	a = 27.567 (1) Å<br>b = 28.289 (5) Å<br>c = 31.738 (8) Å<br>α = 72.680 (1)°<br>β = 76.315 (2)°<br>γ = 79.988(8)°
Volume	22814 (7) Å^3
Z	2
Density (calculated)	0.933 g/cm^3
Absorption coefficient	0.354 μ/mm^{-1}
Colour and shape	Colorless, blocks
Crystal size	0.300 x 0.100 x 0.050 mm
Index ranges	$-23 \leq h \leq 23$<br>$-24 \leq k \leq 24$<br>$-27 \leq l \leq 27$
Reflections collected	138168
R_{int}	0.1195
Data/restraints/parameters	28531/387/3074
Goodness-of-fit on F^2	1.921
Final R indexes [$I > 2\sigma(I)$]	R_1 = 0.1462<br>wR_2 = 0.4031
Final R indexes [all data]	R_1 = 0.1618<br>wR_2 = 0.4263
Largest diff. peak and hole	1.40/-1.19 e Å^{-3}
Total potential solvent accessible void volume from SQUEEZE	8487.6 Å^3
Electron count/cell	3025
CCDC #	2209327

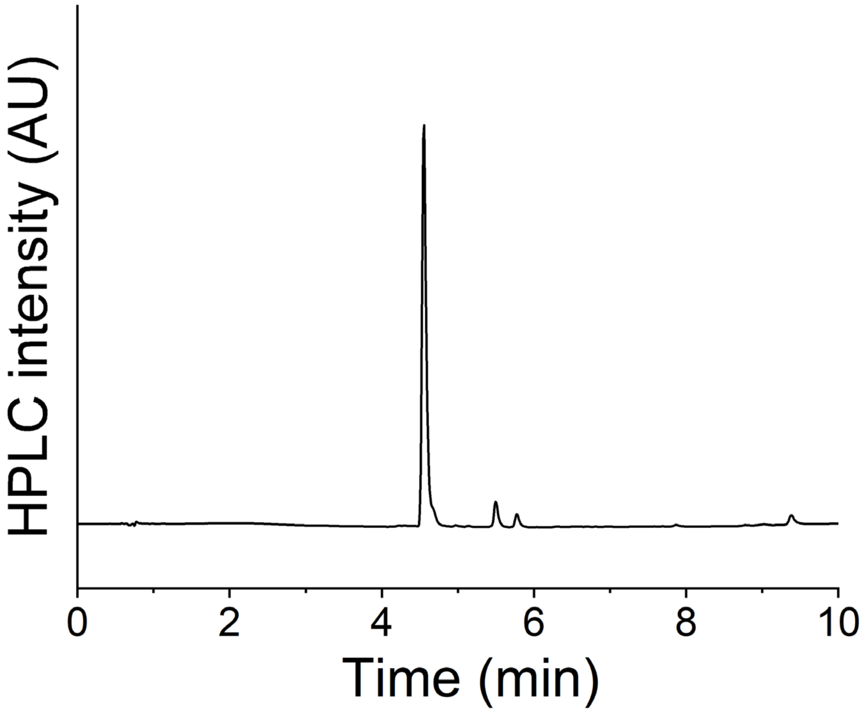

Figure S1. UHPLC analysis of building block **1**.

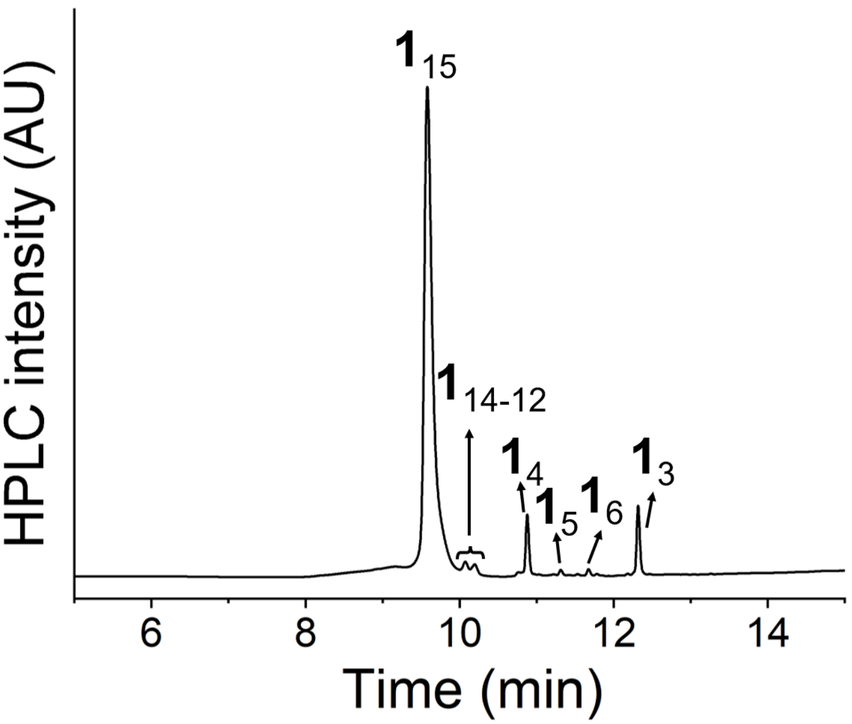

Figure S2. UHPLC-PDA-MS analyses of the DCL made from 1.0 mM building block **1** in 50 mM borate buffer (pH 8.2) without stirring at day 8. Wavelength: 254 nm. The retention times for different compounds are: 9.58 min ($\mathbf{1}_{15}$); 9.96-10.27 min ($\mathbf{1}_{14}$, $\mathbf{1}_{13}$, $\mathbf{1}_{12}$,); 10.88 min ($\mathbf{1}_{4}$); 11.31 min ($\mathbf{1}_{5}$); 11.67 min ($\mathbf{1}_{6}$); 12.32 min ($\mathbf{1}_{3}$).

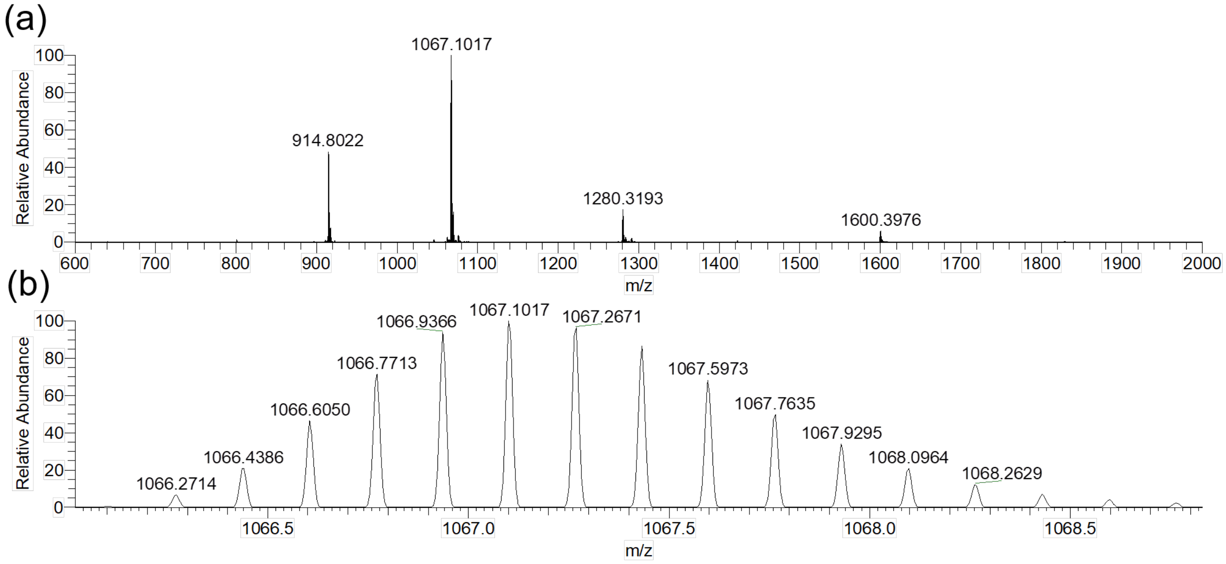

Figure S3. Mass spectrum of **1**$_{15}$ extracted from the UHPLC-PDA-MS analysis shown in Figure S2. (a) Full mass spectrum: m/z observed: 914.8022 $[M+7H]^{7+}$, 1067.1017 $[M+6H]^{6+}$, 1280.3193 $[M+5H]^{5+}$, 1600.3976 $[M+4H]^{4+}$. (b) Isotopic ion peaks of $[M+6H]^{6+}$. Monoisotopic m/z calculated: 1066.2652. Monoisotopic m/z observed: 1066.2714.

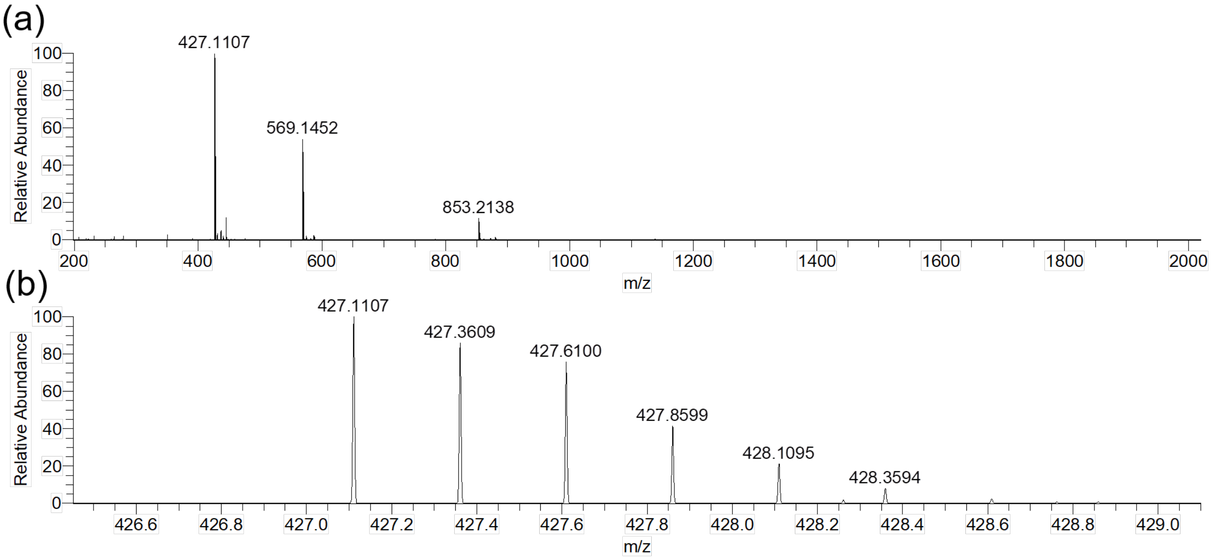

Figure S4. Mass spectrum of $\mathbf{1}_4$ extracted from the UHPLC-PDA-MS analysis shown in Figure S2. (a) Full mass spectrum: m/z observed: 427.1107 $[M+4H]^{4+}$, 569.1452 $[M+3H]^{3+}$, 853.2138 $[M+2H]^{2+}$. (b) Isotopic ion peaks of $[M+4H]^{4+}$. Monoisotopic m/z calculated: 427.1104. Monoisotopic m/z observed: 427.1107.

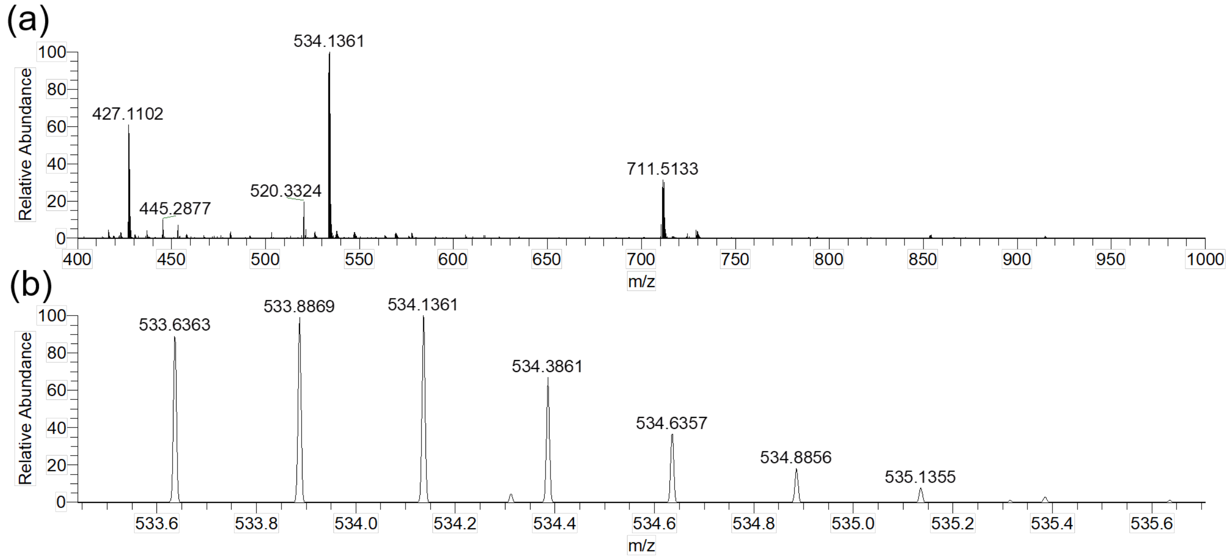

Figure S5. Mass spectrum of $\mathbf{1}_5$ extracted from the UHPLC-PDA-MS analysis shown in Figure S2. (a) Full mass spectrum: m/z observed: 427.1102 $[M+5H]^{5+}$, 534.1361 $[M+4H]^{4+}$, 711.5133 $[M+3H]^{3+}$. (b) Isotopic ion peaks of $[M+4H]^{4+}$. Monoisotopic m/z calculated: 533.6362. Monoisotopic m/z observed: 533.6363.

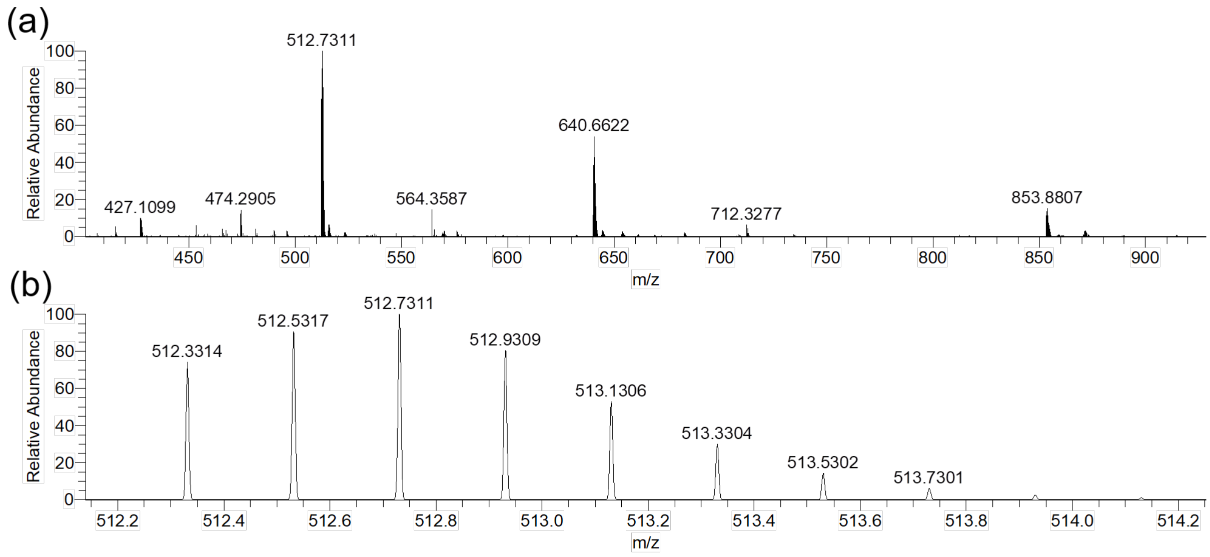

Figure S6. Mass spectrum of $\mathbf{1}_6$ extracted from the UHPLC-PDA-MS analysis shown in Figure S2. (a) Full mass spectrum: m/z observed: 512.7311 $[M+5H]^{5+}$, 640.6622 $[M+4H]^{4+}$, 853.8807 $[M+3H]^{3+}$. (b) Isotopic ion peaks of $[M+5H]^{5+}$. Monoisotopic m/z calculated: 512.3312. Monoisotopic m/z observed: 512.3314.

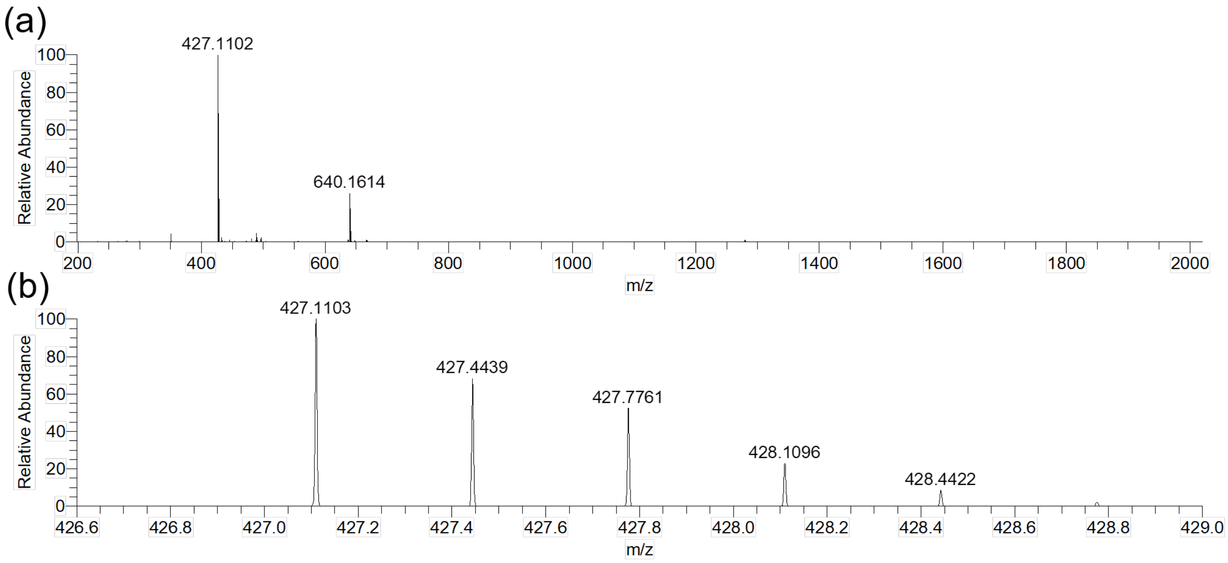

Figure S7. Mass spectrum of $\mathbf{1}_3$ extracted from the UHPLC-PDA-MS analysis shown in Figure S2. (a) Full mass spectrum: m/z observed: 427.1102 $[M+3H]^{3+}$, 640.1614 $[M+2H]^{2+}$. (b) Isotopic ion peaks of $[M+3H]^{3+}$. Monoisotopic m/z calculated: 427.1104. Monoisotopic m/z observed: 427.1103.

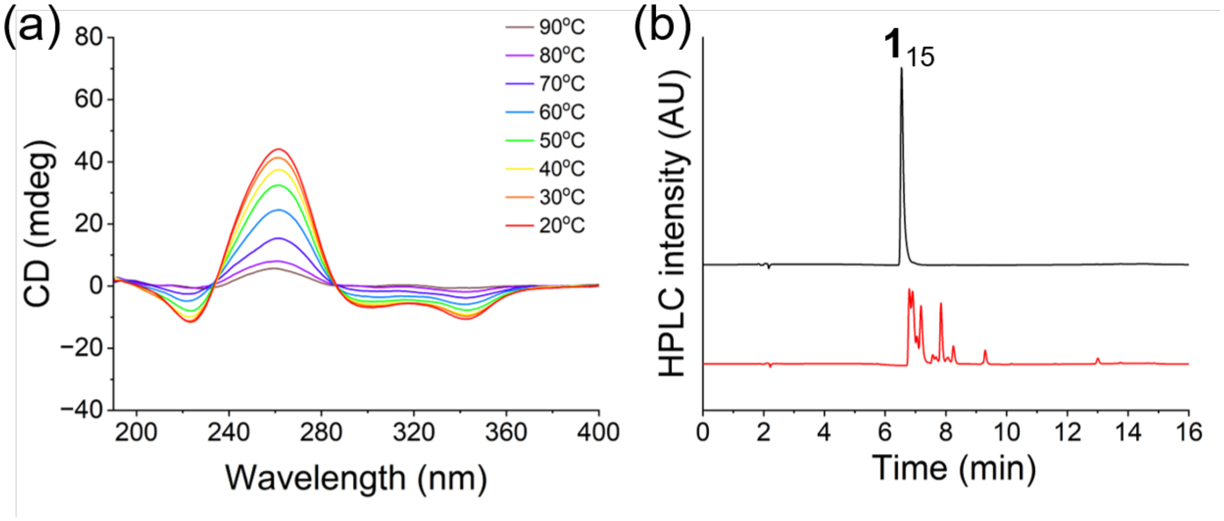

Figure S8. (a) Variable temperature CD spectra of $\mathbf{1}_{15}$ from 90 to 20 °C, (b) HPLC analysis of the $\mathbf{1}_{15}$ sample before (black line) and after (red line) the same heat-cool cycle.

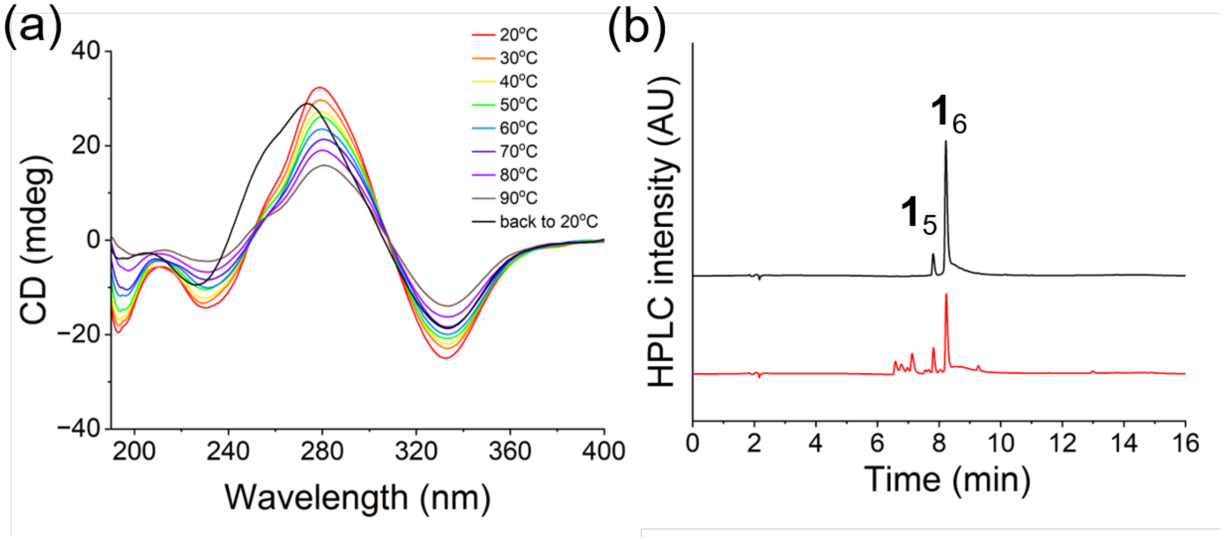

Figure S9. (a) Variable temperature CD spectra of $\mathbf{1}_6$ (20-90-20 °C), (b) HPLC analysis of the $\mathbf{1}_6$ sample before (black line) and after (red line) the same heat-cool cycle.

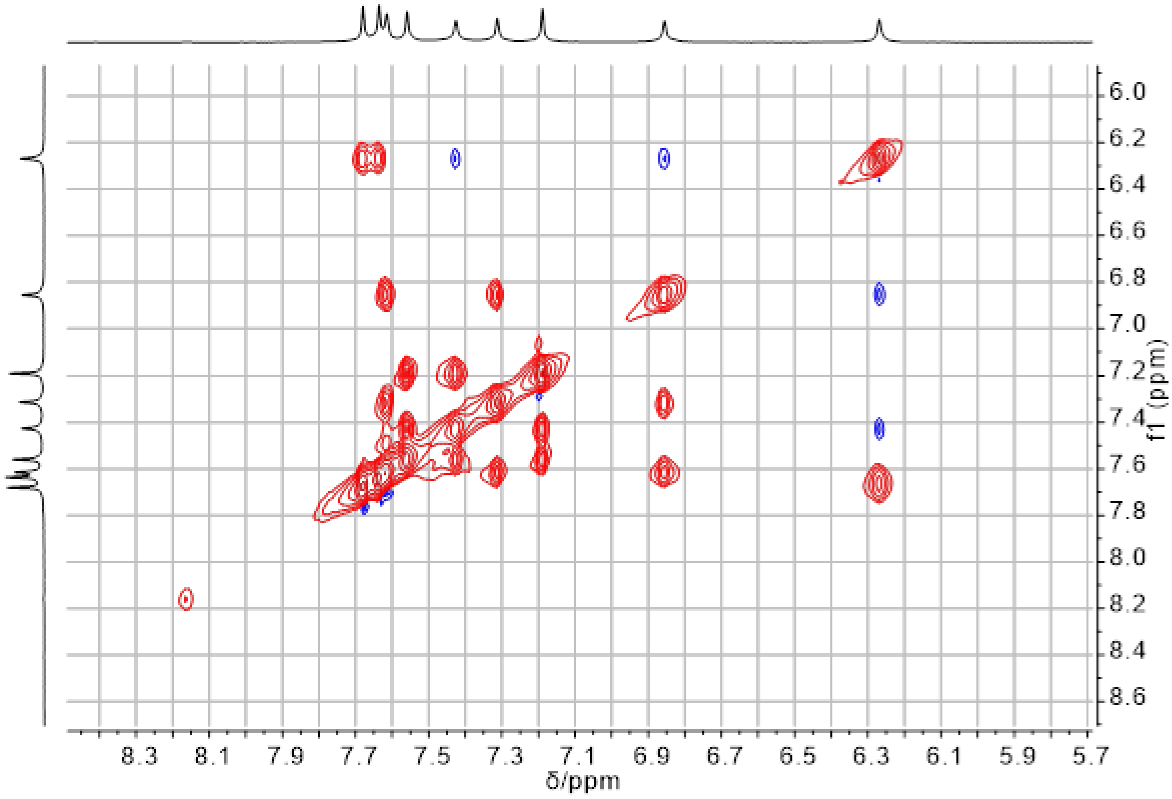

Figure S10. TOCSY spectra of $\mathbf{1}_{15}$.

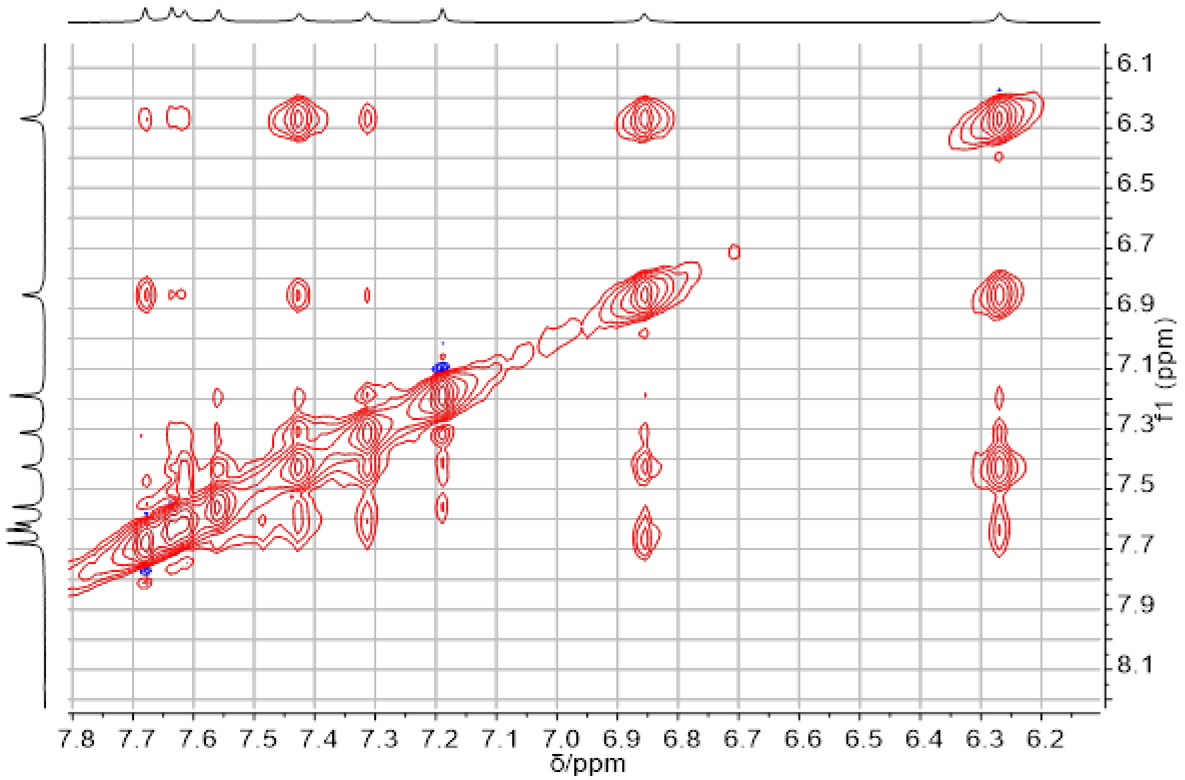

Figure S11. NOESY spectra of $\mathbf{1}_{15}$.

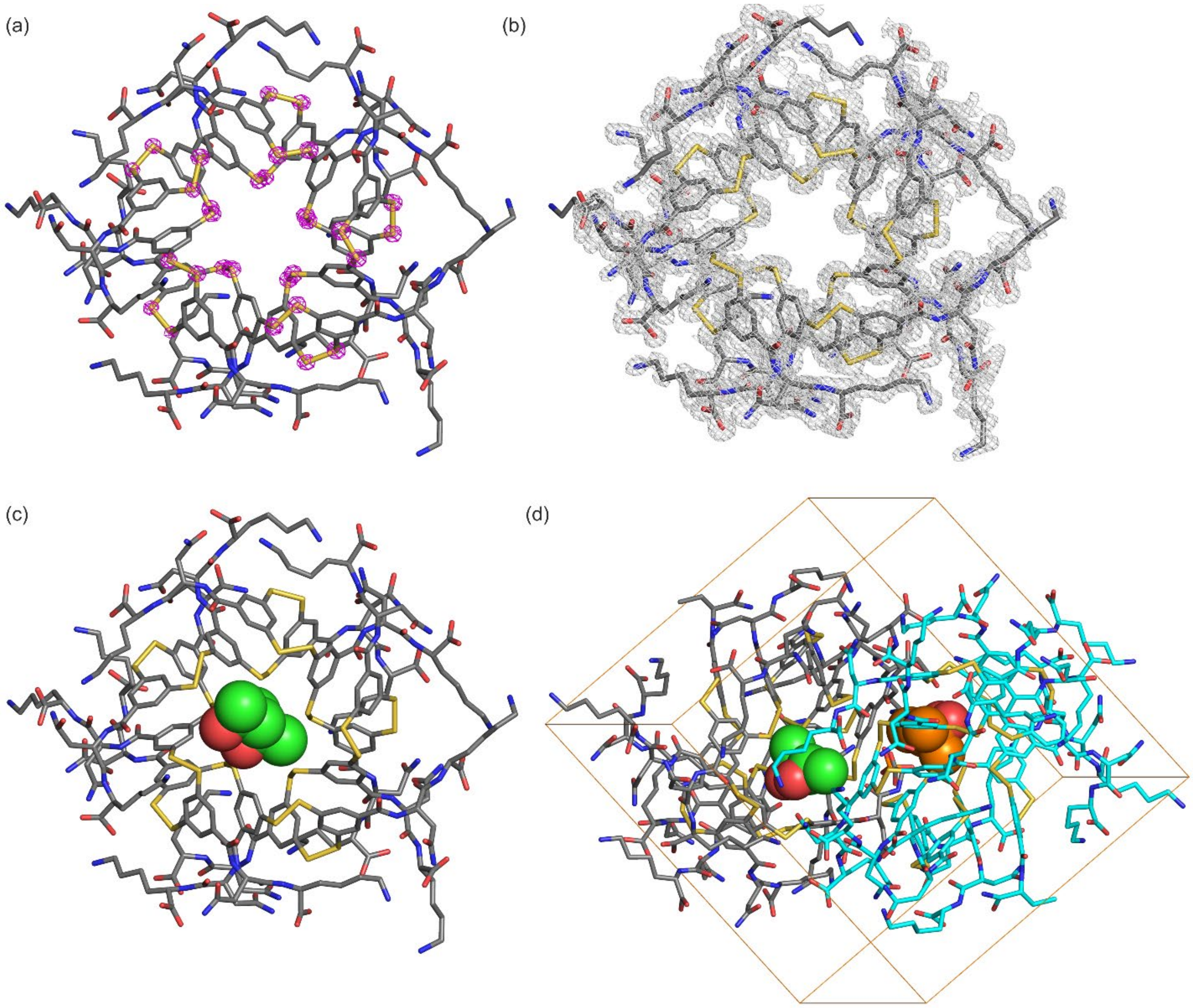

Figure S12. (a, b) Sigma weighted $2F_o$-F_c electron density maps superimposed on L-**1**$_{15}$ macrocycle from the crystal structure of L/D-**1**$_{15}$. (a) Magenta mesh, contoured at 8 σ level shows the position of sulfur atoms and (b) grey mesh, contoured at 1 σ level shows the shape of macrocycle. (c) Space filling representation of the (+/-)-2-methyl-2,4-pentanediol (MPD) molecule (from the crystallization reagent) in the central cavity of the macrocycle. (d) Packing arrangement of L-**1**$_{15}$ (grey) and D-**1**$_{15}$ (cyan) in the unit cell.

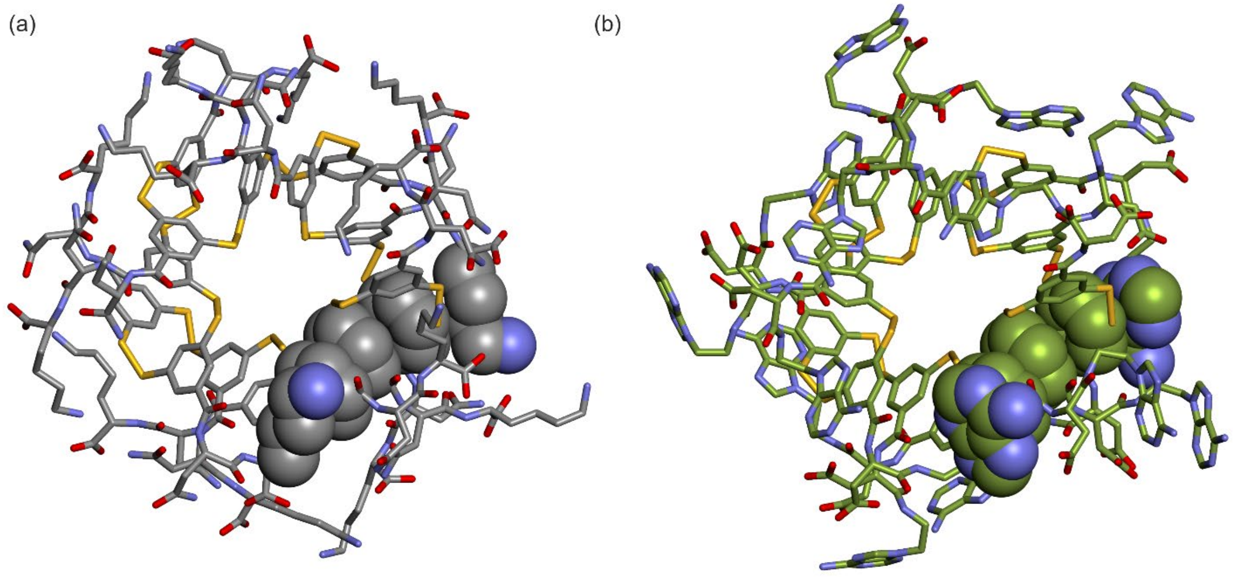

Figure S13. Top view of (a) L-$\mathbf{1}_{15}$ and (b) 15mer of the analogous structure formed from a monomer bearing aspartic acid and adenine, highlighting a stack of three core benzene rings and two lysines/ adenines on the top and bottom of the stack.

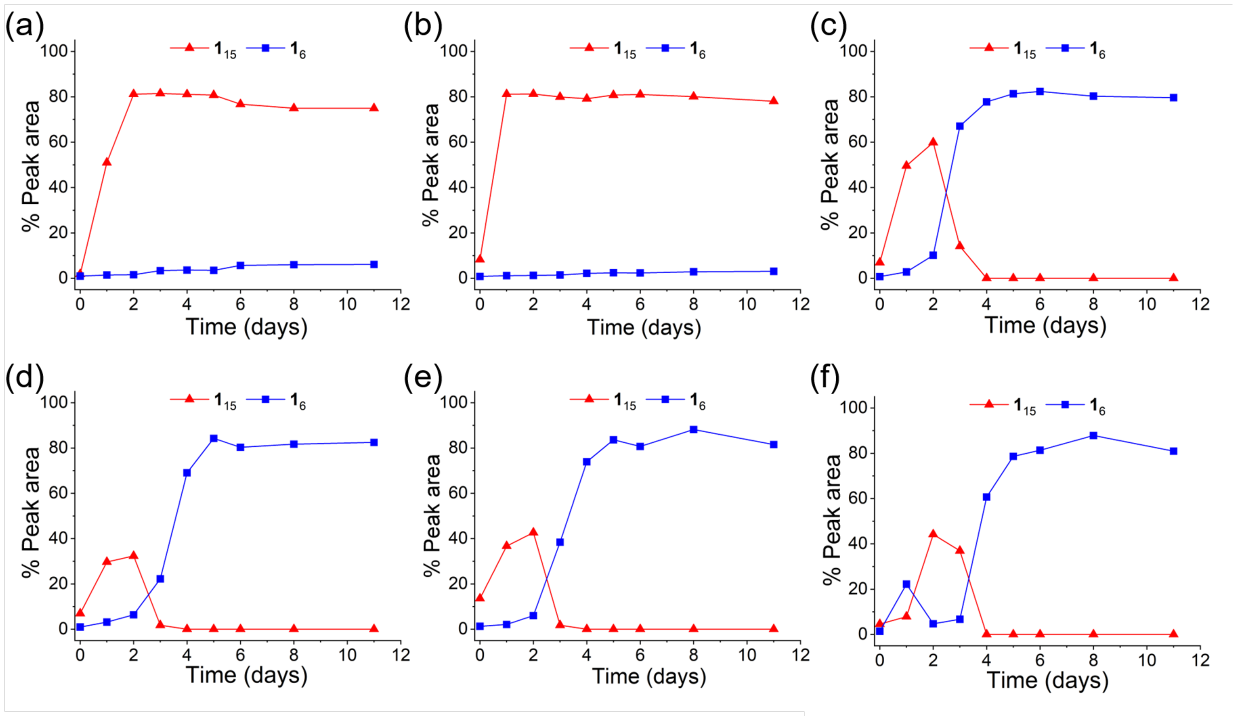

Figure S14. Kinetic profile for DCLs in citrate-phosphate buffer of different pH under magnetic stirring (600 rpm) at (a) pH 3.1; (b) pH 4.1; (c) pH 5.3; (d) pH 6.4; (e) pH 7.3; (f) pH 8.2.

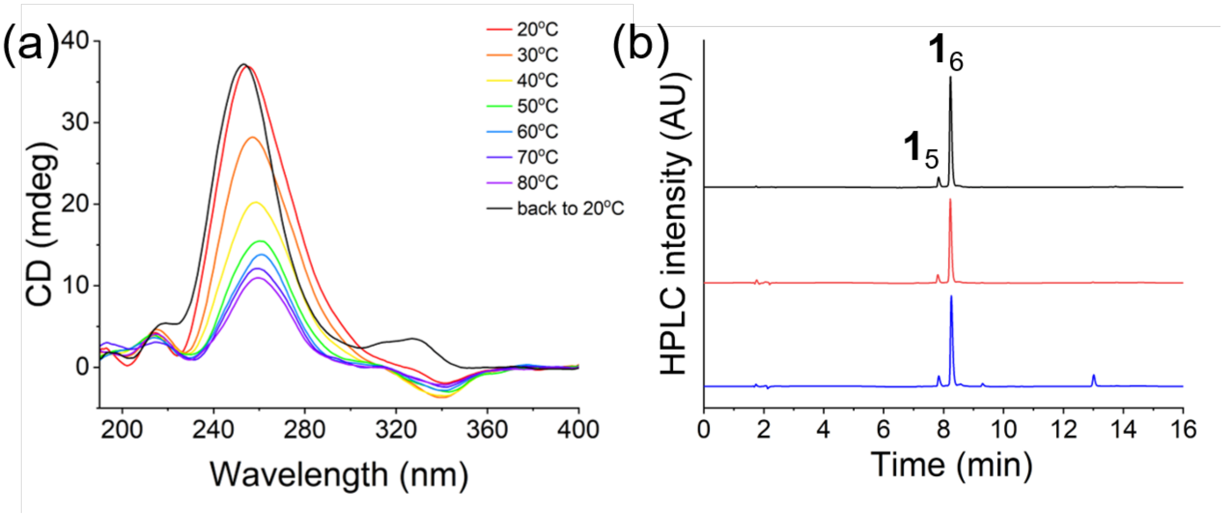

Figure S15. (a) Variable temperature (20-80-20 °C) CD spectra of $\mathbf{1}_6$ in acidic solution (5×10^{-2} M HCl), (b) HPLC analysis of the $\mathbf{1}_6$ sample before adding HCl (black line), after adding HCl (red line), and after the heat-cool cycle of $\mathbf{1}_6$ in acidic solution (5×10^{-2} M HCl; blue line).

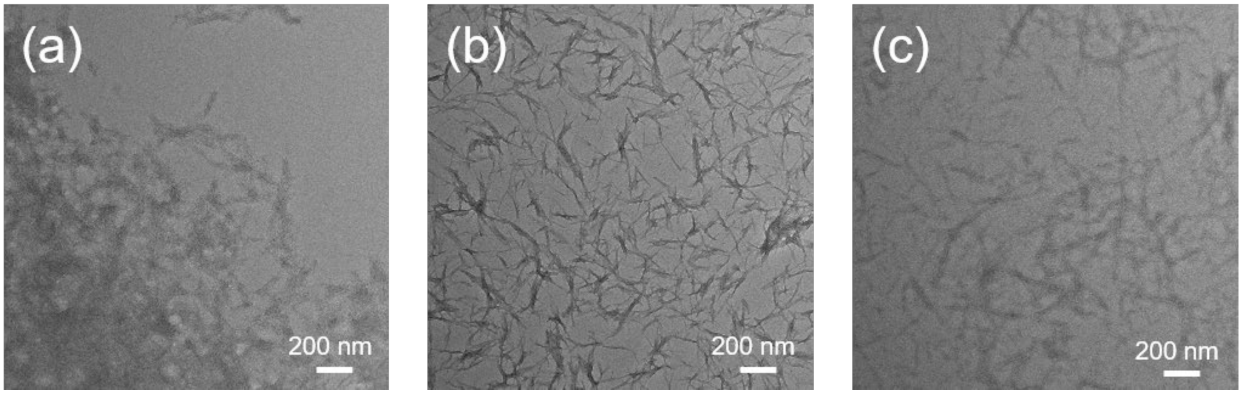

Figure S16. Negative stain TEM micrographs of **1**$_6$ samples at (a) pH 7.3; (b) pH 4.1; (c) pH 1.5.

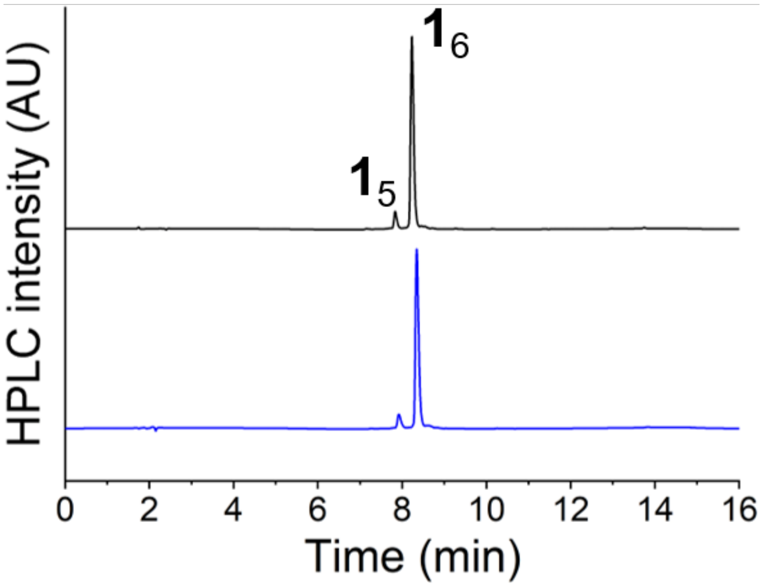

Figure S17. HPLC analysis of the $\mathbf{1}_6$ sample before adding 0.5 M HCl (black line), and 11 days after adding 0.5 M HCl (blue line).

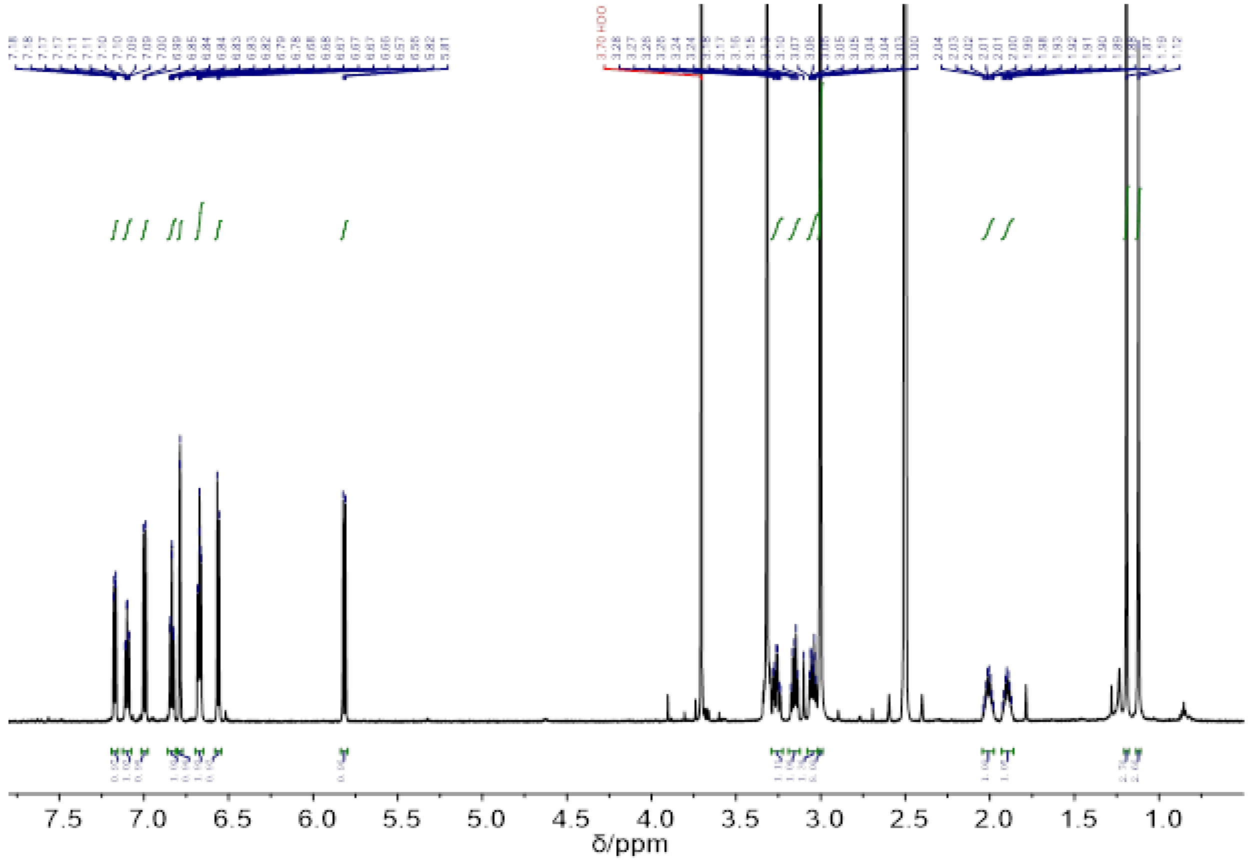

Figure S18. ^{1}H NMR spectra (700 MHz) at 298K of photoacid **3** in DMSO-d_6.

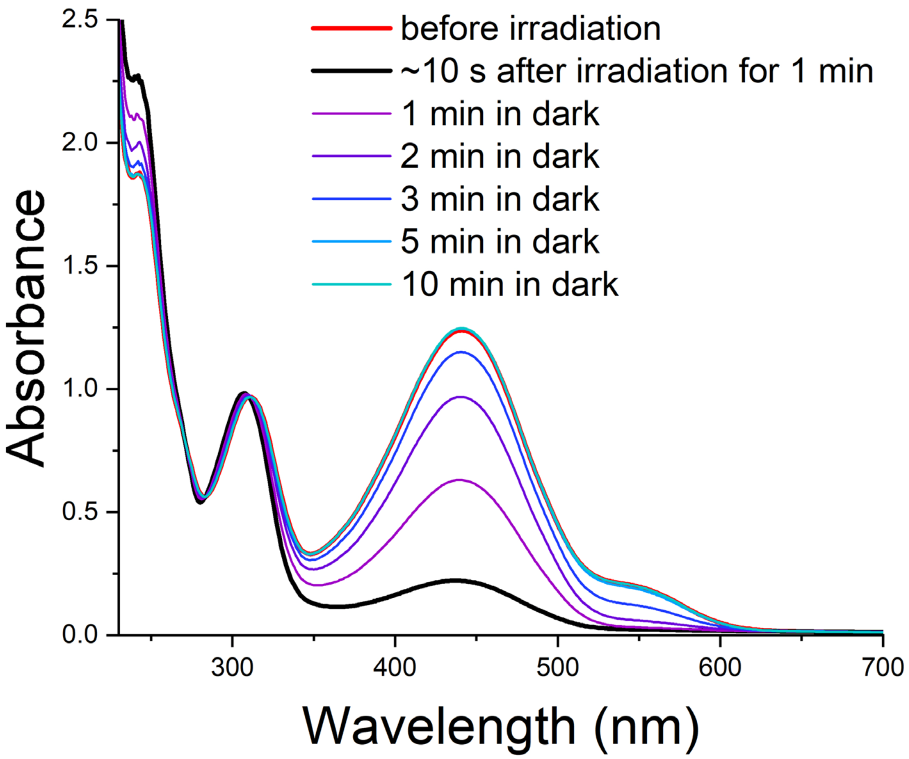

Figure S19. UV-Vis absorption of the photoacid **3** (MCH) solution (0.1 mM in water).

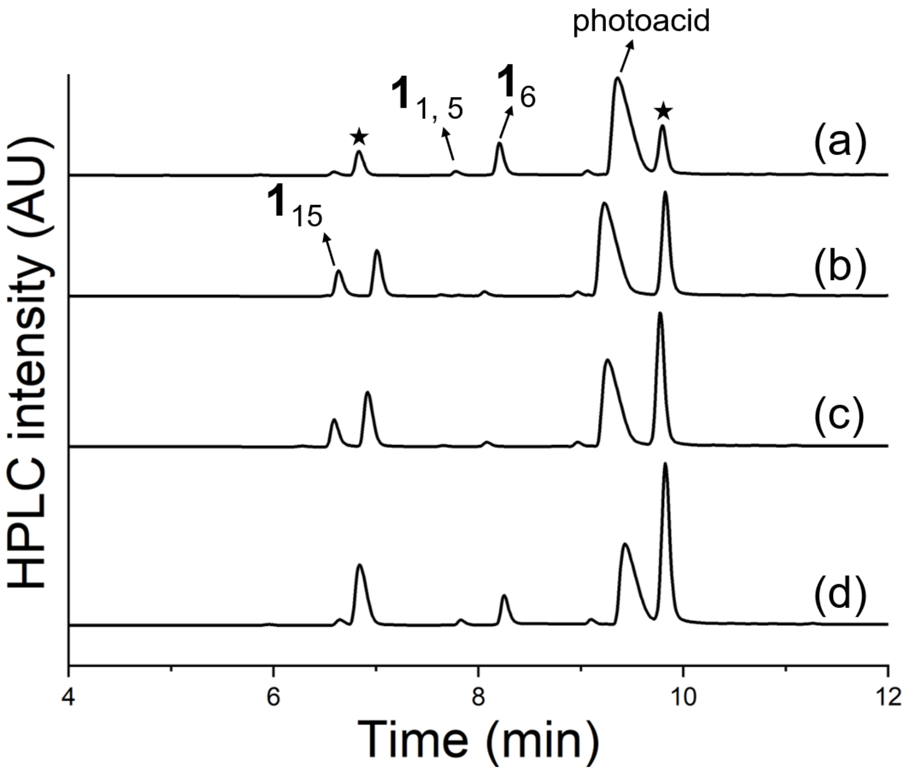

Figure S20. UHPLC chromatograms of DCLs at different times while subjected to a light-dark cycle: (a) 0 h, (b) 2.5 h, (c) 4.0 h, and (d) 16.0 h. Conditions at different time periods: 0-2.0 h, dark and stirred; 2.0-2.5 h, light and stirred; 2.5-4.0 h, dark and not stirred; 4.0-16.0 h, dark, stirred. Peaks marked with a star are the two hydrolyzed products of photoacid **3**.

3. References

(1) Wimberger, L.; Prasad, S. K. K.; Peeks, M. D.; Andreasson, J.; Schmidt, T. W.; Beves, J. E., Large, tunable, and reversible pH changes by merocyanine photoacids. *J. Am. Chem. Soc.* **2021,** *143*, 20758-20768.

(2) Flot, D.; Mairs, T.; Giraud, T.; Guijarro, M.; Lesourd, M.; Rey, V.; van Brussel, D.; Morawe, C.; Borel, C.; Hignette, O.; Chavanne, J.; Nurizzo, D.; McSweeney, S.; Mitchell, E., The ID23-2 structural biology microfocus beamline at the ESRF. *J Synchrotron Radiat* **2010,** *17*, 107-18.

(3) Evans, P., Scaling and assessment of data quality. *Acta Crystallogr. Sect. D-Struct. Biol.* **2006,** *62*, 72-82.

(4) Evans, P. R.; Murshudov, G. N., How good are my data and what is the resolution? *Acta Crystallogr. Sect. D-Biol. Crystallogr.* **2013,** *69*, 1204-1214.

(5) Kabsch, W., Xds. *Acta Crystallogr. Sect. D-Biol. Crystallogr.* **2010,** *66*, 125-132.

(6) Vonrhein, C.; Flensburg, C.; Keller, P.; Sharff, A.; Smart, O.; Paciorek, W.; Womack, T.; Bricogne, G., Data processing and analysis with the autoPROC toolbox. *Acta Crystallogr. Sect. D-Struct. Biol.* **2011,** *67*, 293-302.

(7) Winn, M. D.; Ballard, C. C.; Cowtan, K. D.; Dodson, E. J.; Emsley, P.; Evans, P. R.; Keegan, R. M.; Krissinel, E. B.; Leslie, A. G. W.; McCoy, A.; McNicholas, S. J.; Murshudov, G. N.; Pannu, N. S.; Potterton, E. A.; Powell, H. R.; Read, R. J.; Vagin, A.; Wilson, K. S., Overview of the CCP4 suite and current developments. *Acta Crystallogr. Sect. D-Struct. Biol.* **2011,** *67*, 235-242.

(8) Sheldrick, G. M., SHELXT - Integrated space-group and crystal-structure determination. *Acta Crystallogr. Sect. A* **2015,** *71*, 3-8.

(9) Sheldrick, G. M., Crystal structure refinement with SHELXL. *Acta Crystallogr. Sect. C-Struct. Chem.* **2015,** *71*, 3-8.

(10) Dolomanov, O. V.; Bourhis, L. J.; Gildea, R. J.; Howard, J. A. K.; Puschmann, H., OLEX2: a complete structure solution, refinement and analysis program. *J. Appl. Crystallogr.* **2009,** *42*, 339-341.

(11) Emsley, P.; Lohkamp, B.; Scott, W. G.; Cowtan, K., Features and development of Coot. *Acta Crystallogr. Sect. D-Biol. Crystallogr.* **2010,** *66*, 486-501.

(12) Spek, A. L., Structure validation in chemical crystallography. *Acta Crystallogr. Sect. D-Struct. Biol.* **2009,** *65*, 148-155.
